# Supplementary material for: Efficacy of metamizole versus ibuprofen and a short educational intervention versus standard care in acute and subacute low back pain: a study protocol of a randomised, multicentre, factorial trial (EMISI trial)
Source: BMJ Open. 2021 Oct 13;11(10):e048531. doi: 10.1136/bmjopen-2020-048531 (PMC8515449; doi:10.1136/bmjopen-2020-048531)
Supplement: Author's manuscript [file bmjopen-2020-048531.draft_revisions.pdf]

BMJ Open is committed to open peer review. As part of this commitment we make the peer review history of every article we publish publicly available.

When an article is published we post the peer reviewers' comments and the authors' responses online. We also post the versions of the paper that were used during peer review. These are the versions that the peer review comments apply to.

The versions of the paper that follow are the versions that were submitted during the peer review process. They are not the versions of record or the final published versions. They should not be cited or distributed as the published version of this manuscript.

BMJ Open is an open access journal and the full, final, typeset and author-corrected version of record of the manuscript is available on our site with no access controls, subscription charges or pay-per-view fees (http://bmjopen.bmj.com).

If you have any questions on BMJ Open's open peer review process please email info.bmjopen@bmj.com

BMJ Open

Efficacy of Metamizole versus Ibuprofen and a Short Educational Intervention versus Standard Care in Acute and Subacute Low Back Pain: A Study Protocol of a Randomized, Multicenter, Factorial Trial (EMISI Trial)

Journal:	*BMJ Open*
Manuscript ID	bmjopen-2020-048531
Article Type:	Protocol
Date Submitted by the Author:	02-Jan-2021

Complete List of Authors:	Wertli, Maria M; University of Bern, Department of General Internal Medicine<br>Flury, Julian S.; University of Bern, Department of General Internal Medicine<br>Streit, Sven; Institute of Primary Health Care BIHAM<br>Limacher, Andreas; Universitat Bern Institut fur Sozial- und Praventivmedizin<br>Schuler, Vanessa; University of Bern, Department of General Internal Medicine<br>Ferrante, Asha-Naima; University of Bern, Department of General Internal Medicine<br>Rimensberger, Caroline; University of Bern, Department of General Internal Medicine<br>Haschke, Manuel; University of Bern, Clinical Pharmacology and Toxicology

Keywords:	PAIN MANAGEMENT, Back pain < ORTHOPAEDIC & TRAUMA SURGERY, NEUROPHYSIOLOGY

SCHOLARONE™
Manuscripts

I, the Submitting Author has the right to grant and does grant on behalf of all authors of the Work (as defined in the below author licence), an exclusive licence and/or a non-exclusive licence for contributions from authors who are: i) UK Crown employees; ii) where BMJ has agreed a CC-BY licence shall apply, and/or iii) in accordance with the terms applicable for US Federal Government officers or employees acting as part of their official duties; on a worldwide, perpetual, irrevocable, royalty-free basis to BMJ Publishing Group Ltd ("BMJ") its licensees and where the relevant Journal is co-owned by BMJ to the co-owners of the Journal, to publish the Work in this journal and any other BMJ products and to exploit all rights, as set out in our licence.

The Submitting Author accepts and understands that any supply made under these terms is made by BMJ to the Submitting Author unless you are acting as an employee on behalf of your employer or a postgraduate student of an affiliated institution which is paying any applicable article publishing charge ("APC") for Open Access articles. Where the Submitting Author wishes to make the Work available on an Open Access basis (and intends to pay the relevant APC), the terms of reuse of such Open Access shall be governed by a Creative Commons licence – details of these licences and which Creative Commons licence will apply to this Work are set out in our licence referred to above.

Other than as permitted in any relevant BMJ Author's Self Archiving Policies, I confirm this Work has not been accepted for publication elsewhere, is not being considered for publication elsewhere and does not duplicate material already published. I confirm all authors consent to publication of this Work and authorise the granting of this licence.

For peer review only

Efficacy of Metamizole versus Ibuprofen and a Short Educational Intervention versus Standard Care in Acute and Subacute Low Back Pain: A Study Protocol of a Randomized, Multicenter, Factorial Trial (EMISI Trial)

Maria M. Wertli[1], Julian S. Flury[1], Sven Streit[2], Andreas Limacher[3], Vanessa Schuler[1], Asha-Naima Ferrante[1,4], Caroline Rimensberger[1], Manuel Haschke[5]

[1]Department of General Internal Medicine, Inselspital, Bern University Hospital, University of Bern, Bern, Switzerland

[2]Institute of Primary Health Care (BIHAM), University of Bern, Bern, Switzerland.

[3]CTU Bern, University of Bern, Bern, Switzerland

[4]Faculty of Human Sciences and Department of Psychology, University of Bern, Bern Switzerland

[5]Clinical Pharmacology and Toxicology, Department of General Internal Medicine, Inselspital, Bern University Hospital, University of Bern, Switzerland

Corresponding author

Maria Wertli, MD PhD

Department of General Internal Medicine

Bern University Hospital

Freiburgstrasse 8

3010 Bern

Mail: Maria.Wertli@insel.ch

Phone: +41 31 632 01 46

For peer review only

Abstract

INTRODUCTION Low back pain (LBP) is among the top three most common diseases worldwide, resulting in a life with pain-related disability. To date, no study has assessed the efficacy of metamizole (dipyrone), a non-opioid analgesic and antipyretic prodrug compared to the conventional NSAID ibuprofen, in patients with an acute low back pain episode. Further, it is unclear, whether a short-educational intervention is superior to usual care alone.

OBJECTIVES The objective of this study is to assess first, whether metamizole is non-inferior to ibuprofen in a new episode of acute or subacute LBP. Second, we aim to assess whether a short educational intervention including evidence-based patient information on the nature of LBP is superior to usual care alone.

METHODS AND ANALYSIS An investigator-initiated multicenter, randomized, double blind trial using a factorial design will be performed. A total of 120 participants with a new episode of LBP will be recruited from GP practices, outpatient clinics, and from emergency departments, and randomized into four different treatment groups: ibuprofen alone, ibuprofen and short-intervention, metamizole alone, metamizole and short-intervention. The primary endpoint for the medical treatment will be change in pain assessed on an 11-point numeric rating scale (NRS) after 14 days. The primary outcome for the short intervention will be change in the Core Outcome Index (COMI) assessed after 42 days.

ETHICS, DISSEMINATION, AND FUNDING This study has been approved by the responsible Ethics Board (Ethikkommission Bern / 2018-01986) and the Swiss Agency for Therapeutic Products (Swissmedic / 2019DR4002). Results will be published in open access policy peer-reviewed journals. The study is funded by the Swiss National Science Foundation (grant number 32003B_179346).

For peer review only - http://bmjopen.bmj.com/site/about/guidelines.xhtml

TRIAL REGISTRATION NCT04111315

For peer review only

For peer review only - http://bmjopen.bmj.com/site/about/guidelines.xhtml

Article Summary

Strengths and limitations of this study

- This is the first randomized double-blind study assessing the analgesic effect of metamizole compared to the conventional NSAID ibuprofen in patients with acute or subacute LBP presenting to the GP or emergency department.
- In addition, this study will assess whether a short educational intervention is more effective in LBP patients compared to usual care.
- Due to the high proportion of patients who will not require extended pain treatment during an acute LBP episode, the trial design mirrors current clinical practice.
- The primary endpoint pain intensity will be assessed after 14 days. The primary outcome for the short intervention will be assessed after 42 days.
- While patients, physicians, and researchers are blinded to the pharmacological treatment, blinding is not feasible for the short educational intervention.

Keywords

low back pain, randomized controlled trial, pain medication ibuprofen, NSAID, metamizole, psychological intervention, outcome

Word count:

4983 words.

Protocol Version 1, December 27. 2020

For peer review only - http://bmjopen.bmj.com/site/about/guidelines.xhtml

Introduction

Low back pain (LBP) is among the top three most common diseases worldwide, resulting in a life with pain-related disability.[1] In patients with persistent LBP over 3 months, the risk for chronic pain increases sharply[2] and effective interventions should aim to prevent pain persistence without overtreatment[3]. Therefore, treatment guidelines recommend the use of pain medication and to keep patients physically active during an acute LBP episode.[4 5] Despite the frequency of LBP, only few high-quality studies assessed the efficacy of pain medication. Systematic reviews of randomized clinical trials found opioids to be no more effective than non-opioid medication in LBP[6 7] but opioids were associated with potentially severe side-effects.[4 7 8] Therefore, non-opioid pain medications that are safe and effective are urgently needed to control pain.

Non-steroidal anti-inflammatory drugs (NSAIDs) are effective in LBP treatment and recommended by guidelines.[4 9] However, adverse events including kidney injury and gastrointestinal bleeding limit their use in many patients. Metamizole (dipyrone) is a non-opioid analgesic and antipyretic with a favorable gastrointestinal and renal safety profile.[10] Although Metamizole has been withdrawn from the market or never approved in many countries including the USA, Canada, and UK,[11] it is frequently used in many other countries such as Germany,[12] Spain,[11] Switzerland,[13] some Eastern European, and South American countries.[11 14] Despite its increasing use in many European countries,[13 15] the role of metamizole for the treatment of LBP is unclear and has so far not been systematically studied. This may be due to an ongoing controversy regarding the risk of metamizole-associated agranulocytosis.[11 14] Even though the overall risk of agranulocytosis is increased compared to other analgesics, it remains a rare event occurring only in a small proportion of susceptible patients[16-27] and the overall safety profile of metamizole is still favorable compared to NSAIDs or opioids.[28]

For peer review only - http://bmjopen.bmj.com/site/about/guidelines.xhtml

Many patients have reservations against regular intake of pain medication and, contrary to guideline recommendations, limit physical activity to keep pain manageable without medication.[29] Patient education encouraging physical activity may help to improve pain control and function. For example, a Cochrane review found that 2.5-hour educational sessions were more effective than no intervention on the return-to-work rate in patients with acute/subacute LBP. [30] Such time-intensive interventions may not be necessary in all patients and are not feasible in a busy primary care practice. Short educational interventions would be more practical, however, to date it is unclear whether they also have a positive effect on outcome in patients with a new LBP episode.

Objectives

The first objective of this study is to assess whether metamizole is non-inferior to ibuprofen in patients with a new episode of acute or subacute LBP. The second objective is to assess whether a short educational intervention, including evidence-based patient information on the nature of LBP, is superior to usual care alone. Thus, this randomized trial will provide new knowledge regarding the efficacy of non-opioid pain medications in LBP. In addition, if the short educational intervention proves to be beneficial compared to usual care, the trial will help to improve care for patients with a new LBP episode.

Methods and analysis

The methods reporting of this randomized trial will follow the recommendations of the SPIRIT statement.[31]

Design: Multicenter, randomized, double-blind trial using a factorial design

For peer review only - http://bmjopen.bmj.com/site/about/guidelines.xhtml

The EMISI study is a multicenter, randomized, double-blind trial using a factorial design to investigate whether metamizole is non-inferior to ibuprofen in patients with a new onset LBP episode. Patients, physicians, and researchers will be blinded to the allocation of the study medication. A non-inferiority design was chosen as metamizole seems to be equally effective as ibuprofen in clinical practice. Furthermore, the study will assess whether a short educational intervention including evidence-based patient information is superior to usual care. Blinding patients about the short intervention is not possible but blinding is upheld in GPs and researchers (double blind). The primary evaluation of outcomes (see Outcome measure for details) will be performed at day 14 (comparison of metamizole to ibuprofen) and at day 42 (comparison of educational short intervention to usual care).

Eligibility criteria

Subjects fulfilling all of the following inclusion criteria are eligible for the study:

- Age 18 years or older
- Seeking care for new onset of non-specific or specific LBP (pain duration of less than 12 weeks LBP prior to the baseline visit)
- The GP plans to prescribe a non-opioid pain medication for pain control
- Informed consent documented by signature

Exclusion criteria are:

- Current use of opioids
- Presence of red flags (serious neurological deficit requiring surgery, infection, vertebral fracture)

For peer review only - http://bmjopen.bmj.com/site/about/guidelines.xhtml

- Known intolerance to the study medication (i.e. previous acute allergic reaction to NSAIDs or metamizole)
- Active malignancy and / or history of a (previous) hematologic disorder (history of agranulocytosis)
- History of anemia (hemoglobin of <100.0 mg/L), neutropenia (leucocyte count of < 3.0G/L), thrombocytopenia (<100G/L)
- Known contraindications against the study medications: previous gastrointestinal ulcer/bleeding, inflammatory bowel disease, heart failure (NYHA III-IV), liver failure (liver cirrhosis, ascites), renal insufficiency (estimated glomerular filtration rate (eGFR) < 60 ml/min/1,73 m2) or previous acute kidney injury (AKI stage 2 according to the KDIGO definition)
- Immune deficiency or under immunosuppressant treatment
- Known or suspected non-compliance, drug or alcohol abuse
- Participation in another study within 30 days preceding randomization and during the present study or previous enrolment into the current study
- Enrolment of the investigator, his/her family members, employees, and other dependent persons
- Pregnancy: In women of childbearing age, a negative pregnancy test (urine or blood test as available in the primary care practice) before inclusion is required. Women who are not willing to use safe contraception (condom or birth control pill) during the trial, intention to become pregnant during the trial, pregnancy, or breast feeding.

Study setting

For peer review only - http://bmjopen.bmj.com/site/about/guidelines.xhtml

The EMISI trial will be coordinated by the central study team at the Division of General Internal Medicine, University Hospital Bern, Switzerland. Patients will be recruited from outpatient clinics, from GP practices, and from emergency departments. The overall study duration of 42 days (6 weeks) was chosen because treatment guidelines recommend further assessment of patients for multidisciplinary treatment if LBP persists for more than 6 weeks despite pain medication and the advice to stay active. This is a investigator initiated trial. The funder will have no role in the study design, the trial oversight, data collection, analysis of the study, and publication of the results.

For peer review only

Estimated sample size and power

Both interventions are considered for determining the sample size for this factorial trial.[32] Since there are two primary hypotheses, we adjust the type-I error rate for multiple testing by setting the alpha level to 0.025 in each test, keeping the overall type-I error rate at 0.05. In order to achieve the two objectives of the study, at least 120 patients with a new onset LBP episode will be randomly allocated to one of the four treatment groups.

We consider a difference in the change of pain score in the numeric rating scale (NRS; range 0 to 10, higher score indicates more pain) of two or more score points between the two groups as clinically relevant. A change of 1 score point or less is regarded as negligible.[33 34] Therefore, we set the non-inferiority margin to -1 score point. Based on a two-sample means test and a standard deviation of 1.6,[33] we will need 108 patients (54 per group) to proof non-inferiority of the metamizole treatment at a one-sided alpha-level of 2.5% with a power of 90%.

The sample size for the primary outcome comparison short intervention vs. usual care (superiority) is based on a change in Core Outcome Measures Index (COMI; range 0 to

For peer review only - http://bmjopen.bmj.com/site/about/guidelines.xhtml

10, higher score indicates higher level of complaint, see primary outcomes) from baseline to week 6. We hypothesize that there is a difference between the two intervention groups regarding COMI. Mannion et al.[35] reported a minimal clinically important difference for improvement in the COMI of 2.2 points and standard deviations for changes in COMI ranging from 1.7 to 2.5 points. We see a difference of 2.2 points between the two groups as clinically relevant and assume a standard deviation of 2.2 points. Based on a two-sample means test, we will need 54 patients (27 per group) to detect a difference in the change of the COMI at a two-sided alpha-level of 2.5% with a power of 90%. To account for a drop-out of 10% we aim to include 120 patients.

Primary outcome

Table 1 provides an overview of measurements according to the Standard Protocol Items: Recommendations for Interventional Trials. The primary outcome for the comparison of metamizole vs. ibuprofen is the change in pain on the NRS (range 0-10 points) from baseline to follow-up at day 14 (non-inferiority). Pain intensity will be recorded as average pain intensity for the past 24 hours on an NRS scale from 0 to 10, scored from 0 (no pain) to 10 (worst possible pain). The rational for assessing the effectiveness outcome at 14 days was based on the double-blind RCT comparing paracetamol to placebo.[36]

The primary outcome for the comparison of the short intervention vs. usual care is the change in the COMI sum-score (range 0-10) from baseline to 42 days follow-up (superiority). The COMI is a short, multidimensional, validated outcome instrument with excellent psychometric properties recommended for the monitoring of the outcome in patients undergoing treatment for LBP.[35] The COMI sum score is calculated as described in the validation paper.[37 38] The pain scales (item 2a and 2b) are scored 0-10,

For peer review only - http://bmjopen.bmj.com/site/about/guidelines.xhtml

whilst the category scales (items 3-7) are scored as 0, 2.5, 5.0, 7.5, 10.0. The sum score (0-10) for the whole COMI scale is calculated based on the following 5 domains:

- Pain domain: higher of the two pain scores (higher of 2a and 2b) is taken as the score

- Disability domain: the average of the two disability items (6 and 7)

- Back related function (item 3)

- Symptom specific well-being (item 4)

- General quality of life (item 5)

The five domain scores are then averaged to give a COMI score from 0-10.

Two additional questions cover the satisfaction with treatment outcome. A minimal clinically important difference (MCID) found to be relevant for patients was a reduction in COMI score of ≥2.2 points.[35]

Secondary outcome

All outcome measures used in this study were recommended by the NIH Task force and other expert panels to study back pain.[39] [40] **Table 1** provides an overview of measurements according to the Standard Protocol Items: Recommendations for Interventional Trials.[31]

Secondary outcome measures include:

- Change in pain on the numeric rating scale (NRS, range 0-10 points) from baseline to follow-up at day 42
- Change in the Core Outcome Measures Index (COMI) sum-score (range 0-10) from baseline to 14 days follow-up
- The proportion of patients with a minimal clinically important difference (MCID) in the NRS (≥2 points) and COMI (≥2.2 points) at days 14 and 42

For peer review only - http://bmjopen.bmj.com/site/about/guidelines.xhtml

- Time to recovery (recovery defined as the first day with a NRS of <2 points sustained for 2 consecutive days)
- Change in overall pain intensity from baseline to follow-up at day 14 and day 42. Overall pain intensity: average of ratings for average pain, pain at rest, and pain during activity during the last 24 hours.
- Area under effect curve (AUEC day 0 to day 14) NRS
- Adverse events (AE)
- Time to stopping treatment
- Global effectiveness of the treatment (COMI question ‘‘how much did the operation/treatment help your back problem?’’ 5-point Likert scale from ‘‘helped a lot’’ to ‘‘made things worse’’)
- Daily physical activity (daily step count)
- Use of pain medication and additional rescue medication
- Additional treatment (physical therapy, injections)
- Return to work (yes / no)
- Treatment satisfaction (COMI)
- Quality of life as assessed by the 5-level version of the European Quality of Life-5 Dimensions questionnaire (EQ-5D)
- Psychological factors (STarT-Back Tool)

Explanatory variables

We will collect blood samples during visit 2 (day 14) to assess the following factors that may influence the individual response to the pain medications. First, we will measure

For peer review only - http://bmjopen.bmj.com/site/about/guidelines.xhtml

plasma concentrations of ibuprofen and the four main metamizole metabolites (4-MAA, 4-AA, 4-FA, 4-AAA). Pharmacodynamic effects of metamizole correlate primarily with concentrations of the 4-MAA metabolite,[41] thus direct quantification of 4-MAA concentrations will add important information for the interpretation of the observed effects.[42]

Second, as individual response to ibuprofen may be affected by genetic polymorphisms of CYP isoforms 2C8 and 2C9, the study population will be characterized in terms of cytochrome P450 (CYP) 2C8/9 genotypes (CYP2C8 *1 and *3 alleles, CYP2C9 *1, *2, and *3 alleles). In subjects with alleles conferring low CYP2C8 and / or CYP2C9 activity, reduced ibuprofen clearance of both enantiomers has been described, resulting in several-fold increased exposure and prolonged half-life compared to subjects with wild-type alleles.[43 44]

Study procedures

Study procedures are summarized in the study flow chart (**Figure 1**). Each participating site will receive a supply of sequentially coded, blinded, and sealed intervention packs. All patients presenting with LBP will be screened for potential inclusion into the study. After obtaining informed consent by the physician, patient information will be entered in a web-based clinical data management system (CDMS). After patient registration in the CDMS and confirmation of all inclusion and exclusion criteria, patients are randomized using sequentially coded and blinded intervention packs, thus ensuring concealment of allocation. The number of the intervention pack will be recorded in the CDMS. After this, patients may open the intervention pack and start treatment.

For peer review only - http://bmjopen.bmj.com/site/about/guidelines.xhtml

The intervention pack will contain a pill bottle and an envelope with the assignment of the short vs no intervention, and a pain diary. Patients will record intake of study drug and additional pain medication in the pain diary. To quantify physical activity during the study, all patients will receive a commercially available wearable step tracking device (Garmin vivofit 4®) and enter the daily step count in the pain diary. Data collection includes self-reported patient characteristics using standardized and validated questionnaires, the clinical assessment by the GPs, and a blood sample for serum creatinine and a differential blood count.

To mitigate the risk for AEs, we will collect blood samples after the randomization in all patients. In case of a newly detected renal insufficiency (defined as estimated glomerular filtration rate (eGFR) < 60 ml/min/1.73 m^2), leucopenia (leucocyte count < 3.0G/L), thrombocytopenia (defined as <100 G/L) and/or anemia ((hemoglobin <100g/L), we will stop the study medication.

During the second visit (day 14), patients will return the pill bottles and the pain diary to the GPs office where they are collected for pill count. The patient will receive a new drug pack and pain diary. A blood sample will be collected (creatinine, blood count, drug concentrations and genotyping), and self-reported questionnaires and a clinical assessment will be completed.

To improve adherence, study staff will call patients between the first and the second visit and between the second and the final visit and ask for adverse events and additional pain treatment. As we expect a high proportion of patients to have recovered by day 28 (second phone call), researchers will then determine whether a clinical visit on day 42 is necessary. In case patients have recovered by day 28, patients do not need to attend the clinical visit.

For peer review only - http://bmjopen.bmj.com/site/about/guidelines.xhtml

A final assessment will be done on day 42 during a clinical visit in patients who had persisting symptoms on day 28. Patients will return the pain diary and the pill bottles with the remaining pills to the GPs office where they are collected for pill count. Data collection includes again self-reported validated questionnaires, and the clinical assessment by the GPs. A final blood sample (serum creatinine and blood count) will only be collected if patients are still taking study medication. Patients who do not need to attend the clinical visit will receive a final identical set of questionnaires by mail and will be asked for AE/SAE and additional treatments during a phone call. Patients will return the questionnaires together with the pain diary and the pill bottles to the central study team. To assess the success of blinding, patients will be asked at the end of the study what treatment group they believe they had been assigned to (metamizole or ibuprofen).

Pharmacological intervention

During the first four days, we will compare a fixed-dose regimen of metamizole 500mg to 500 mg ibuprofen lysine (lysine-2-[4-isobutylphenyl]-propionate, corresponds to 292.6 mg ibuprofen acid) three times two capsules per day (**Table 2**). Ibuprofen was chosen as a comparator because it is a well-studied and the most frequently prescribed NSAID in Switzerland[13] with a similar dosing interval as metamizole. The initial dose of both medications is the currently used and recommended dose for the treatment of acute pain (common daily dose for metamizole 3000mg (maximum daily dose 4000mg) and 3000mg ibuprofen lysine (maximum daily dose 4000mg)). Ibuprofen lysine 3000mg corresponds to 1800mg ibuprofen acid. The ibuprofen lysine formulation was chosen to facilitate blinding of the study drug, as ibuprofen lysine 500mg was the only ibuprofen formulation available on the Swiss market, which could be prescribed with identical number of capsules and at identical intervals as metamizole. The lysine salt of ibuprofen

For peer review only - http://bmjopen.bmj.com/site/about/guidelines.xhtml

shows better solubility and faster absorption compared with the acid form [41 45]. However, overall extent of absorption and analgesic effect in an acute pain model did not differ from the acid form.[46]

In LBP, current clinical practice is to prescribe an initial fixed dose regimen followed by an as-needed regimen that allows patients to adjust the daily dose depending on their pain intensity. To mirror clinical practice, patients will be allowed after the first four days to decrease the intake of the study medication according to a dose reduction schedule (as needed phase). From day 4 until recovery patients may reduce the daily pain medication intake once their pain has decreased to less than 4 points on the NRS (range 0-10 points) every two days. The treatment duration will thus depend on the duration of pain. Patients are considered to have recovered when their pain is below 2 (NRS 0-10) on 2 consecutive days after they have stopped intake of pain medication (end of treatment).

Non-pharmacological educational short intervention

Patients assigned to the short intervention will receive together with the pain medication a short leaflet focusing on the nature of non-specific LBP and the importance of staying active. A second leaflet contains exercises to alleviate LBP. The content of the short information leaflet is based on the *The Back Book*.[47] Further, all patients assigned to the short intervention will receive a 10-minute standardized telephone intervention delivered by a trained member of the study team during the first 4 treatment days to help patients understand the non-dangerous nature of LBP, and to explain the purpose of pain medication to facilitate movement. In addition, patients will be encouraged to stay active and to perform the exercises as proposed in the exercise leaflet.

Concomitant interventions

For peer review only - http://bmjopen.bmj.com/site/about/guidelines.xhtml

In a double-blind RCT comparing paracetamol to placebo for LBP in the primary care setting, rescue medications were infrequently used.[36] Therefore, physicians will be allowed to prescribe additional rescue medications recommended by treatment guidelines at their discretion. Additional medication use is recorded. Further, information on any use of physical therapy, massage or other treatments is collected during the follow-up visits at days 14 and 42. Although GPs will be instructed to withhold physical therapy until day 14 (i.e. the primary effectiveness endpoint for metamizole vs. ibuprofen) if the clinical situation allows, additional care including physical therapy and relaxation strategies recommended by guidelines will be at the discretion of the treating GP. Concomitant treatments will be treated as co-variates to assess their potential impact on overall efficacy (performance bias).

Randomization and allocation procedures

The allocation sequence (randomization list) will be generated by an independent data manager at the CTU Bern who is not otherwise involved in the trial. Randomization will be blocked and stratified according to study sites. To ensure concealment of allocation, block size will not be communicated. After patient data is registered in the CDMS, eligibility is confirmed and signed informed consent is obtained, patients will be assigned to the next sequentially coded intervention pack that is available at the recruiting site. Patients are randomized 1:1:1:1 into one of four groups: Metamizole + educational intervention vs. metamizole + standard care vs. ibuprofen + educational intervention vs. ibuprofen + standard care. The intervention packs will contain a pill bottle and an envelope with the assignment of the short vs. no intervention. The intervention packs will be prepared according to the randomization list by an independent hospital pharmacist not involved in the study.

Blinding

For peer review only - http://bmjopen.bmj.com/site/about/guidelines.xhtml

Staff at the hospital pharmacy not involved in patient recruitment or other trial activities will fill the gelatin capsules with the study medication and label the pill bottles according to a standard operating procedure and based on the randomization list provided by the CTU Bern. The study medication will be delivered in identically labeled sealed pill bottles containing capsules of identical appearance. The randomization list will be kept electronically at the CTU Bern in a password-protected file. Study personnel and physicians involved in patient recruitment, treatment, and outcome assessment will have no access to the list.

Due to the nature of the short educational intervention, no blinding of patients receiving an information leaflet and a telephone call is possible. Therefore, contamination and performance bias are important to consider. The envelope including the leaflet will be sealed and the patients will be instructed to open de envelope outside the GPs office. GPs will not be informed about the group assignment and the content and aim of the educational intervention. Patients assigned to the educational intervention will receive a telephone call according to a predefined script by a member of the study team otherwise not involved in patient recruitment, outcome assessment, and data analysis. To minimize the risk of detection bias, the study outcomes are collected in pain diaries and self-reported questionnaires (NRS at day 14, COMI at day 42). Study personnel that will contact patients by phone for reminders and to collect information on adverse events will be blinded to the group assignment. To assess differences in additional treatments (performance bias), patients and GPs will record additional treatments other than the study medication and the educational information (e.g. additional medication use, physical therapy, injections).

The statistician responsible for the final analysis will also have no access to the list until the primary analysis of the trial is finished. Data analysis will be done according to a prespecified analysis plan before the randomization code will be broken. Patients,

For peer review only - http://bmjopen.bmj.com/site/about/guidelines.xhtml

physicians, and data analysts will be blinded with regard to the allocation to the study medication until after the primary analysis. To assess the success of blinding, we will ask patients at the end of the study into which group they belief they had been assigned to. Data on all recruited patients will be collected and regularly monitored.

Statistical analysis plan

The statistical analysis will be performed in compliance with the International Conference on Harmonisation's Good Clinical Practice guidelines and the report will be developed in line with the Consolidated Standards of Reporting Trials statement.

To assess the efficacy for the two primary outcomes, we will use two analysis sets (see **Figure 1**). The full analysis set (FAS) includes all randomized patients for the intention-to-treat (ITT) analysis, i.e. all patients will be analyzed in the group they were assigned to. The *per protocol (PP) set* is based on the FAS, excluding patients with major protocol violations, patients who were excluded due to the predefined stop criteria, patients withdrawing from the trial within 3 days of treatment start, patients undergoing surgery, and patients that did not receive the randomized treatment or intervention.

We will present patient and procedural characteristics using descriptive statistics (mean and standard deviation or median and interquartile range for continuous variables and frequency and percentage for categorical variables).

To compare the efficacy of metamizole and ibuprofen (non-inferiority), we use an ITT (FAS set) and a PP analysis (PP set) for the primary outcome (NRS change from baseline to follow-up at 14 days). Both analyses need to meet non-inferiority in order to claim success. The difference in the change in NRS between the two groups will be calculated from a linear model, adjusted for the baseline NRS value and the type of intervention (short intervention vs standard care). If the lower one-sided 97.5% confidence limit of the difference lies above the non-inferiority margin of -1, non-

For peer review only - http://bmjopen.bmj.com/site/about/guidelines.xhtml

inferiority will be claimed. If we can demonstrate non-inferiority, we will additionally test for superiority at a two-sided significance level of 2.5%. Since testing is nested (i.e. hierarchical), this procedure has no implication on the overall type-I error rate.

To compare the primary outcome between the short educational intervention to usual care (superiority), we use ITT analysis (COMI change from baseline to day 42). The difference in the change in COMI between the two groups will be calculated and tested at a two-sided significance level of 2.5% using a linear model, adjusted for the baseline COMI and the type of drug treatment. Differences will be presented with a two-sided 97.5% confidence interval (CI) and the regular 95% CI.

We will assess both primary outcomes for interaction. We will introduce an interaction term between the treatment indicator and the intervention indicator to test whether in A) the effect of the metamizole treatment on the change in NRS at day 14 is different in patients that receive the short intervention compared with the standard care group and in B) whether the effect of the short intervention on the change in COMI at day 42 is different in patients that receive metamizole compared with patients that receive ibuprofen.

Secondary continuous outcomes will be analyzed as the primary outcomes. Besides the indicator for the drug treatment or intervention, models will be adjusted for the baseline value if available. Secondary binary outcomes will be analyzed using logistic regression models adjusted for the indicator for the drug treatment or intervention, as applicable. Time-to-event outcomes will be analyzed using Cox regression adjusted for the indicator for the drug treatment or intervention, as applicable. For all secondary effect measures, we will display regular two-sided 95% CI and accompanying p-values.

Further, we will perform additional PP analyses for the primary outcome of the comparison between the two intervention arms as well as for all secondary outcomes. If

For peer review only - http://bmjopen.bmj.com/site/about/guidelines.xhtml

outcomes are missing, we will employ multiple imputation in the primary analysis and additionally perform an available case analysis disregarding missing data. Continuous outcomes that are measured several times during follow-up will additionally be evaluated in a repeated measures mixed-effects linear model, additionally introducing a random intercept for patients into the model.

Exploratory analysis will be conducted with regards to predictors for treatment responses. We will explore the association between clinical predictors (e.g. depression, fear avoidance beliefs, social factors), drug levels, or genetic mutations and the treatment response in linear models. We will perform subgroup analyses according to gender, genetic phenotypes, and drug levels.

Safety outcomes will be reported as proportion and 95% CI separately for the two drug treatment groups using the safety population (i.e. all patients that have received at least one dose of the study drug and at least one safety-related visit or observation) set.

Patients with missing outcome data will be imputed using multiple imputation.

Safety

Protocol violations should not lead to treatment discontinuation unless they indicate a significant risk to patient safety.

Patients may be withdrawn from the study treatment for the following reasons:

- Hematologic disorders including leucopenia (defined as leucocyte count < 3.0G/L; thrombocytopenia (defined as <100 G/L), and anemia (decrease in hemoglobin ≥20g/L or <100g/L)
- Potential evidence for a hematologic disease (pathological blood smear needing further evaluation)

For peer review only - http://bmjopen.bmj.com/site/about/guidelines.xhtml

- Decreased kidney function (defined as an estimated creatinine clearance using the MDRD formula of < 50 ml/min/1.73m^2) or acute kidney injury stage 1 or higher (defined according to the KDIGO definition: ≥1.5 times creatinine increase compared to baseline value or ≥26.5 micro mol/L creatinine increase)
- Development of toxicity which, in the investigators or GPs judgment, precludes further use of one of the study drugs
- Patients lost to follow-up or non-compliance

We will comply with all regulations concerning safety measures in clinical trials as set forth by the Swiss Agency for Therapeutic Products (Swissmedic). The investigators will report any serious adverse events occurring during clinical trials, independent of direct causal relationship with the treatment, within 24 hours. In case of a suspected unexpected serious adverse reaction (SUSAR), Swissmedic will be notified by the sponsor-investigator within the legal timelines. Unblinding will be permissible in case the information is required to ensure the patients safety in case of an AE.

All adverse events (AE) are reviewed by a safety committee and graded according to the National Health Service (NIH) Common Terminology Criteria for Adverse Events (CTCAE v5.0).[48]

Patient and public involvement

Patients will be asked during the phone call on day 28 whether they would be willing to participate in an in-person interview at the end of the study. The aim of the interview is to assess factors that were relevant for patients during their LBP treatment. The interview will be recorded (video and audio) for qualitative analysis using standard grounded theory method according to Philipp Mayring.[49]

For peer review only - http://bmjopen.bmj.com/site/about/guidelines.xhtml

Ethics and dissemination

The trial will be performed in compliance with the Declaration of Helsinki and its amendments, and has been approved by the responsible Ethics Board (Ethikkommission Bern/ 2018-01986) and the Swiss Agency for Therapeutic Products (Swissmedic / 2019DR4002). Important protocol modifications will be communicated to the Ethics Board and participating study sites. Potential participants must provide written informed consent before entering the study. Subjects can leave the study at any time for any reason without any negative consequences for their further treatment. When a patient ends the treatment phase of the study prematurely, we will record the date and reason for early treatment discontinuation. If possible, the end of treatment evaluations will be collected before the patient is started on any other therapeutic intervention. Insurance coverage will be provided for all study participants by the study sponsor.

The results of the main trial and each of the secondary outcomes will be submitted for publication in open access peer-reviewed journals. Results will be disseminated to all participating centers and participants expressing interest. The EMISI study group is an independent academic research group, which will not employ professional writers. The EMISI study group will comply with the open access regulation of the Swiss National Science Foundation.

For peer review only

Authors' contributions

MMW, SS, AL, JF, and MH designed the study. MMW and JF wrote the first draft of the manuscript. All authors revised the manuscript and approved the final version.

For peer review only - http://bmjopen.bmj.com/site/about/guidelines.xhtml

Funding statement

Efficacy of Metamizole versus Ibuprofen and a Short Educational Intervention versus Standard Care in Acute and Subacute Low Back Pain: A Randomized, Factorial Trial (The EMISI Trial) is supported by the Swiss National Science Foundation (grant number: 32003B_179346 / 1). This research project received no further support from any other funding agency.

Competing interests

There is no conflict of interest regarding intellectual content and proprietary affairs.

Data monitoring committee

Data monitoring will be conducted by the CTU Bern. Data collection, handling of the data, and the analysis will be done by the researchers at the University of Bern. The funder plays no role in the data acquisition, data storage, and analysis.

There is no interim analysis planned. Auditing will be performed by an independent auditor of the CTU at the central study site.

Trial sponsor investigator

Maria Wertli, MD PhD

Department of General Internal Medicine

Bern University Hospital

Freiburgstrasse 8

3010 Bern

Mail: Maria.Wertli@insel.ch

Phone: +41 31 632 01 46

For peer review only - http://bmjopen.bmj.com/site/about/guidelines.xhtml

Bibliography

1. Vos T, Allen C, Arora M, et al. Global, regional, and national incidence, prevalence, and years lived with disability for 310 diseases and injuries, 1990-2015: a systematic analysis for the Global Burden of Disease Study 2015. *Lancet (London, England)* 2016;388(10053):1545-602. doi: 10.1016/s0140-6736(16)31678-6 [published Online First: 2016/10/14]
2. da C Menezes Costa L, Maher CG, Hancock MJ, et al. The prognosis of acute and persistent low-back pain: a meta-analysis. *CMAJ: Canadian Medical Association journal* 2012;184(11):E613-E24. doi: 10.1503/cmaj.111271 [published Online First: 05/14]
3. Itz CJ, Geurts JW, van Kleef M, et al. Clinical course of non-specific low back pain: a systematic review of prospective cohort studies set in primary care. *European journal of pain (London, England)* 2013;17(1):5-15. doi: 10.1002/j.1532-2149.2012.00170.x [published Online First: 2012/05/30]
4. Qaseem A, Wilt TJ, McLean RM, et al. Noninvasive treatments for acute, subacute, and chronic low back pain: A clinical practice guideline from the american college of physicians. *Annals of internal medicine* 2017;166(7):514-30. doi: 10.7326/M16-2367
5. van Tulder M, Becker A, Bekkering T, et al. Chapter 3. European guidelines for the management of acute nonspecific low back pain in primary care. *European spine journal : official publication of the European Spine Society, the European Spinal Deformity Society, and the European Section of the Cervical Spine Research Society* 2006;15 Suppl 2:S169-91. doi: 10.1007/s00586-006-1071-2 [published Online First: 2006/03/22]
6. Chou R, Deyo R, Friedly J, et al. Systemic Pharmacologic Therapies for Low Back Pain: A Systematic Review for an American College of Physicians Clinical Practice Guideline. *Annals of internal medicine* 2017;166(7):480-92. doi: 10.7326/m16-2458 [published Online First: 2017/02/14]
7. Chaparro LE, Furlan AD, Deshpande A, et al. Opioids compared with placebo or other treatments for chronic low back pain: an update of the Cochrane Review. *Spine* 2014;39(7):556-63. doi: 10.1097/brs.0000000000000249 [published Online First: 2014/02/01]
8. Hoffman EM, Watson JC, St Sauver J, et al. Association of Long-term Opioid Therapy With Functional Status, Adverse Outcomes, and Mortality Among Patients With Polyneuropathy. *JAMA Neurol* 2017;74(7):773-79. doi: 10.1001/jamaneurol.2017.0486
9. Wong JJ, Cote P, Sutton DA, et al. Clinical practice guidelines for the noninvasive management of low back pain: A systematic review by the Ontario Protocol for Traffic Injury Management (OPTIMa) Collaboration. *European journal of pain (London, England)* 2017;21(2):201-16. doi: 10.1002/ejp.931 [published Online First: 2016/10/07]
10. Konijnenbelt-Peters J, van der Heijden C, Ekhart C, et al. Metamizole (Dipyrone) as an Alternative Agent in Postoperative Analgesia in Patients with Contraindications for Nonsteroidal Anti-Inflammatory Drugs. *Pain practice : the official journal of World Institute of Pain* 2017;17(3):402-08. doi: 10.1111/papr.12467 [published Online First: 2016/06/28]
11. Stammschulte T, Ludwig WD, Muhlbauer B, et al. Metamizole (dipyrone)-associated agranulocytosis. An analysis of German spontaneous reports 1990-2012. *European journal of clinical pharmacology* 2015;71(9):1129-38. doi: 10.1007/s00228-015-1895-y [published Online First: 2015/07/15]

For peer review only - http://bmjopen.bmj.com/site/about/guidelines.xhtml

12. Hoffmann F, Meinecke P, Freitag MH, et al. Who gets dipyrone (metamizole) in Germany? Prescribing by age, sex and region. *Journal of clinical pharmacy and therapeutics* 2015;40(3):285-8. doi: 10.1111/jcpt.12261 [published Online First: 2015/03/18]
13. Wertli MM, Reich O, Signorell A, et al. Changes over time in prescription practices of pain medications in Switzerland between 2006 and 2013: an analysis of insurance claims. *BMC health services research* 2017;17(1):167. doi: 10.1186/s12913-017-2086-6
14. Hoffmann F, Bantel C, Jobski K. Agranulocytosis attributed to metamizole: An analysis of spontaneous reports in EudraVigilance 1985-2017. *Basic & Clinical Pharmacology & Toxicology* 2020;126(2):116-25. doi: https://doi.org/10.1111/bcpt.13310
15. Hoffmann F, Schmiemann G. Pain medication in German nursing homes: a whole lot of metamizole. *Pharmacoepidemiology and drug safety* 2016;25(6):646-51. doi: 10.1002/pds.3954 [published Online First: 2016/01/11]
16. Basak GW, Drozd-Sokolowska J, Wiktor-Jedrzejczak W. Update on the incidence of metamizole sodium-induced blood dyscrasias in Poland. *The Journal of international medical research* 2010;38(4):1374-80. doi: 10.1177/147323001003800419
17. Blaser LS, Tramonti A, Egger P, et al. Hematological safety of metamizole: retrospective analysis of WHO and Swiss spontaneous safety reports. *European journal of clinical pharmacology* 2015;71(2):209-17. doi: 10.1007/s00228-014-1781-z
18. Hamerschlak N, Maluf E, Biasi Cavalcanti A, et al. Incidence and risk factors for agranulocytosis in Latin American countries--the Latin Study: a multicenter study. *European journal of clinical pharmacology* 2008;64(9):921-9. doi: 10.1007/s00228-008-0513-7 [published Online First: 2008/06/27]
19. Huber M, Andersohn F, Sarganas G, et al. Metamizole-induced agranulocytosis revisited: results from the prospective Berlin Case-Control Surveillance Study. *European journal of clinical pharmacology* 2015;71(2):219-27. doi: 10.1007/s00228-014-1777-8
20. Ibanez L, Vidal X, Ballarin E, et al. Population-based drug-induced agranulocytosis. *Archives of internal medicine* 2005;165(8):869-74. doi: 10.1001/archinte.165.8.869
21. Ibanez L, Vidal X, Ballarin E, et al. Agranulocytosis associated with dipyrone (metamizol). *European journal of clinical pharmacology* 2005;60(11):821-9. doi: 10.1007/s00228-004-0836-y [published Online First: 2004/12/08]
22. Kramer M, Lane D, Hutchinson T. Risks of agranulocytosis and aplastic anemia. A first report of their relation to drug use with special reference to analgesics. The International Agranulocytosis and Aplastic Anemia Study. *Jama* 1986;256(13):1749-57.
23. Maj S, Centkowski P. A prospective study of the incidence of agranulocytosis and aplastic anemia associated with the oral use of metamizole sodium in Poland. *Medical science monitor : international medical journal of experimental and clinical research* 2004;10(9):Pi93-5. [published Online First: 2004/08/26]
24. Maj S, Lis Y. The incidence of metamizole sodium-induced agranulocytosis in Poland. *The Journal of international medical research* 2002;30(5):488-95. doi: 10.1177/147323000203000504 [published Online First: 2002/11/27]
25. Shapiro S, Issaragrisil S, Kaufman DW, et al. Agranulocytosis in Bangkok, Thailand: a predominantly drug-induced disease with an unusually low incidence. Aplastic Anemia Study Group. *The American journal of tropical medicine and hygiene* 1999;60(4):573-7. [published Online First: 1999/05/29]

For peer review only - http://bmjopen.bmj.com/site/about/guidelines.xhtml

26. van der Klauw MM, Goudsmit R, Halie MR, et al. A population-based case-cohort study of drug-associated agranulocytosis. *Archives of internal medicine* 1999;159(4):369-74. [published Online First: 1999/02/25]
27. Varonos DD, Santamouris S, Karambali S. The incidence of dipyrone-induced agranulocytosis in Greece during 1975. *The Journal of international medical research* 1979;7(6):564-8. doi: 10.1177/030006057900700617
28. Kötter T, da Costa BR, Fässler M, et al. Metamizole-Associated Adverse Events: A Systematic Review and Meta-Analysis. *PloS one* 2015;10(4):e0122918. doi: 10.1371/journal.pone.0122918
29. Rosser BA, McCracken LM, Velleman SC, et al. Concerns about medication and medication adherence in patients with chronic pain recruited from general practice. *Pain* 2011;152(5):1201-5. doi: 10.1016/j.pain.2011.01.053 [published Online First: 2011/03/08]
30. Engers A, Jellema P, Wensing M, et al. Individual patient education for low back pain. *The Cochrane database of systematic reviews* 2008(1):Cd004057. doi: 10.1002/14651858.CD004057.pub3 [published Online First: 2008/02/07]
31. Chan AW, Tetzlaff JM, Gøtzsche PC, et al. SPIRIT 2013 explanation and elaboration: guidance for protocols of clinical trials. *BMJ (Clinical research ed)* 2013;346:e7586. doi: 10.1136/bmj.e7586 [published Online First: 2013/01/11]
32. Chow SC, Shao J, Wang H. Sample Size Calculations in Clinical Research. 2nd ed. New York: Chapman & Hall/CRC 2008.
33. Farrar JT, Young JP, Jr., LaMoreaux L, et al. Clinical importance of changes in chronic pain intensity measured on an 11-point numerical pain rating scale. *Pain* 2001;94(2):149-58.
34. Ostelo RW, Deyo RA, Stratford P, et al. Interpreting change scores for pain and functional status in low back pain: towards international consensus regarding minimal important change. *Spine* 2008;33(1):90-4. doi: 10.1097/BRS.0b013e31815e3a10
35. Mannion AF, Porchet F, Kleinstuck FS, et al. The quality of spine surgery from the patient's perspective: part 2. Minimal clinically important difference for improvement and deterioration as measured with the Core Outcome Measures Index. *European spine journal : official publication of the European Spine Society, the European Spinal Deformity Society, and the European Section of the Cervical Spine Research Society* 2009;18 Suppl 3:374-9. doi: 10.1007/s00586-009-0931-y
36. Williams CM, Maher CG, Latimer J, et al. Efficacy of paracetamol for acute low-back pain: a double-blind, randomised controlled trial. *Lancet (London, England)* 2014;384(9954):1586-96. doi: 10.1016/s0140-6736(14)60805-9 [published Online First: 2014/07/30]
37. Mannion AF, Elfering A, Staerkle R, et al. Outcome assessment in low back pain: how low can you go? *European spine journal : official publication of the European Spine Society, the European Spinal Deformity Society, and the European Section of the Cervical Spine Research Society* 2005;14(10):1014-26. doi: 10.1007/s00586-005-0911-9 [published Online First: 2005/06/07]
38. Pochon L, Kleinstuck FS, Porchet F, et al. Influence of gender on patient-oriented outcomes in spine surgery. *European spine journal : official publication of the European Spine Society, the European Spinal Deformity Society, and the European Section of the Cervical Spine Research Society* 2016;25(1):235-46. doi: 10.1007/s00586-015-4062-3 [published Online First: 2015/07/06]
39. Deyo RA, Dworkin SF, Amtmann D, et al. Report of the NIH Task Force on research standards for chronic low back pain. *Pain medicine (Malden, Mass)* 2014;15(8):1249-67. doi: 10.1111/pme.12538

For peer review only - http://bmjopen.bmj.com/site/about/guidelines.xhtml

40. Pincus T, Santos R, Breen A, et al. A review and proposal for a core set of factors for prospective cohorts in low back pain: a consensus statement. *Arthritis Rheum* 2008;59(1):14-24. doi: 10.1002/art.23251
41. Weiser T, Schepers C, Mück T, et al. Pharmacokinetic Properties of Ibuprofen (IBU) From the Fixed-Dose Combination IBU/Caffeine (400/100 mg; FDC) in Comparison With 400 mg IBU as Acid or Lysinate Under Fasted and Fed Conditions—Data From 2 Single-Center, Single-Dose, Randomized Crossover Studies in Healthy Volunteers. *Clinical Pharmacology in Drug Development* 2019;8(6):742-53. doi: 10.1002/cpdd.672
42. Pierre SC, Schmidt R, Brenneis C, et al. Inhibition of cyclooxygenases by dipyrone. *British journal of pharmacology* 2007;151(4):494-503. doi: 10.1038/sj.bjp.0707239 [published Online First: 2007/04/17]
43. García-Martín E, Martínez C, Tabarés B, et al. Interindividual variability in ibuprofen pharmacokinetics is related to interaction of cytochrome P450 2C8 and 2C9 amino acid polymorphisms. *Clinical Pharmacology & Therapeutics* 2004;76(2):119-27. doi: 10.1016/j.clpt.2004.04.006
44. Kirchheiner J, Meineke I, Freytag G, et al. Enantiospecific effects of cytochrome P450 2C9 amino acid variants on ibuprofen pharmacokinetics and on the inhibition of cyclooxygenases 1 and 2. *Clinical Pharmacology & Therapeutics* 2002;72(1):62-75. doi: 10.1067/mcp.2002.125726
45. Klueglich M, Ring A, Scheuerer S, et al. Ibuprofen extrudate, a novel, rapidly dissolving ibuprofen formulation: relative bioavailability compared to ibuprofen lysinate and regular ibuprofen, and food effect on all formulations. *Journal of clinical pharmacology* 2005;45(9):1055-61. doi: 10.1177/0091270005279579
46. Kyselovič J, Koscova E, Lampert A, et al. A Randomized, Double-Blind, Placebo-Controlled Trial of Ibuprofen Lysinate in Comparison to Ibuprofen Acid for Acute Postoperative Dental Pain. *Pain and Therapy* 2020;9(1):249-59. doi: 10.1007/s40122-019-00148-1
47. Burton AK, Waddell G, Tillotson KM, et al. Information and advice to patients with back pain can have a positive effect. A randomized controlled trial of a novel educational booklet in primary care. *Spine* 1999;24(23):2484-91. [published Online First: 2000/01/08]
48. U.S. Department of health and human services. Common Terminology Criteria for Adverse Events (CTCAE). In: U.S.Department of health and human services NIoH, national Cancer Institute, ed. June 14, 2010 ed: U.S.Department of health and human services,, 2009.
49. Mayring P. Qualitative Inhaltsanalyse. Grundlagen und Techniken. 7. ed. Weinheim: Deutscher Studien Verlag 2000.

For peer review only

For peer review only - http://bmjopen.bmj.com/site/about/guidelines.xhtml

Figure Title and Legend

Figure 1. Study flow chart

For peer review only

For peer review only - http://bmjopen.bmj.com/site/about/guidelines.xhtml

Tables

Table 1: Summary of study procedures based on Standard Protocol Items: Recommendations for Interventional Trials (SPIRIT) schedule

	Enrollment	Allocation	Short intervention	Phone call	Clinical visit	Phone call	Clinical visit or phone call
**Time point (days)**	-5 to 0	0	2	7	14	28	42
Clinical visit	1	1			2		**3 or ✆[4]**
Phone call			✆[2]	✆[3]		✆[3]	
Patient information & informed consent	x						
In-/Exclusion	x						
Demographics	x						
Medical History	x						
Physical examination	x						
Vital signs	x				x		(x)
Blood analysis	x				x		(x)
Pregnancy test	x						
Randomization		x					
Hand out study medication		x					
Daily intake of study medication		x					
Group 1 + 3: study medication only		x		x	x	x	x
Group 2 + 4: study medication + short intervention		x	x	x	x	x	x
Pain diary daily including:<br>- Pain<br>- additional treatments<br>- AE<br>- Daily physical activity		x	x	x	x	x	x
Adherence reminder[3]				x		x	

For peer review only - http://bmjopen.bmj.com/site/about/guidelines.xhtml

Screening for: [9]<br>- AE and SAE<br>- interrupting or discontinuing treatment				x	x	x	x
Collection of pill bottles for pill-count					x		x
Self-reported measures collected during visits							
Pain questionnaire (NRS)		x			x		x
COMI		x			x		x
STarT Back tool		x			x		x
Quality of life (EQ5-D-5L)		x			x		x
Fear avoidance (FABQ)		x					
Self-efficacy (FESS)		x					
Blinding control							x
Voluntary in-person interview on patient perspective							x

Legend: AE, adverse event; SAE, serious adverse event; NRS, numeric rating scale; COMI, Core Outcome Measures Index; EQ5-D-5L, European Quality of Life-5 Dimensions questionnaire; FABQ, Fear Avoidance Questionnaire, FESS, self-efficacy (Schmerzspezifische Selbstwirksamkeit)

For peer review only - http://bmjopen.bmj.com/site/about/guidelines.xhtml

Table 2: Oral doses for study medication – fixed dose and as-needed regimen

Group	Study medication	Fixed dose regimen (days 0 - 3)	As-needed regimen (after day 3): recommended reduction of the oral intake when LBP decreases
1	Ibuprofen only	500mg§ 2-2-2	2-2-2 (pain unchanged), 1-1-1 (decreased pain; NRS <4 points), 1-0-1, 1-0-0
2	Ibuprofen + short intervention	500mg§ 2-2-2	2-2-2 (pain unchanged), 1-1-1 (decreased pain; NRS <4 points), 1-0-1, 1-0-0
3	Metamizole only	500mg 2-2-2	2-2-2 (pain unchanged), 1-1-1 (decreased pain; NRS <4 points), 1-0-1, 1-0-0
4	Metamizole + short intervention	500mg 2-2-2	2-2-2 (pain unchanged), 1-1-1 (decreased pain; NRS <4 points), 1-0-1, 1-0-0

§500 mg ibuprofenum lysinum (lysine-2-[4-isobutylphenyl]-propionate) corresponds to 292.6 mg ibuprofen acid.

For peer review only

Abbreviations

AE	Adverse Event
CDMS	Clinical Data Management System
COMI	Core Outcome Measures Index
CRF	Case Report Form
EMISI	Efficacy of Metamizole versus Ibuprofen and a Short Educational Intervention versus Standard Care
GP	General Physician
ITT	Intention To Treat
LBP	Low Back Pain
NRS	Numeric Rating Scale
NSAIDs	Non-Steroidal Anti-Inflammatory Drugs

For peer review only

For peer review only - http://bmjopen.bmj.com/site/about/guidelines.xhtml

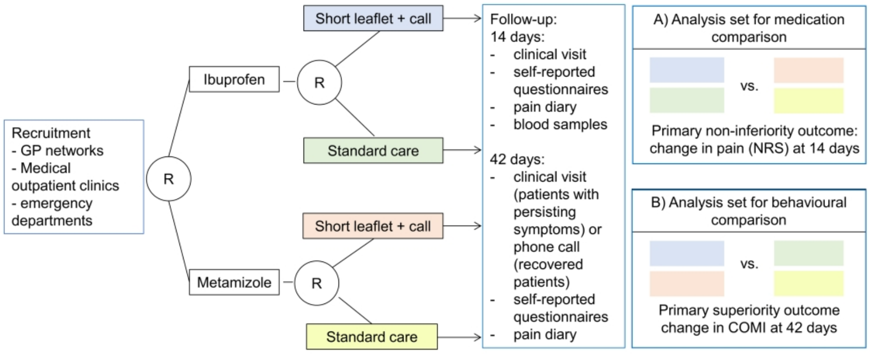

338x190mm (300 x 300 DPI)

Reporting checklist for protocol of a clinical trial.

Based on the SPIRIT guidelines.

Instructions to authors

Complete this checklist by entering the page numbers from your manuscript where readers will find each of the items listed below.

Your article may not currently address all the items on the checklist. Please modify your text to include the missing information. If you are certain that an item does not apply, please write "n/a" and provide a short explanation.

Upload your completed checklist as an extra file when you submit to a journal.

In your methods section, say that you used the SPIRITreporting guidelines, and cite them as:

Chan A-W, Tetzlaff JM, Gøtzsche PC, Altman DG, Mann H, Berlin J, Dickersin K, Hróbjartsson A, Schulz KF, Parulekar WR, Krleža-Jerić K, Laupacis A, Moher D. SPIRIT 2013 Explanation and Elaboration: Guidance for protocols of clinical trials. BMJ. 2013;346:e7586

		Reporting Item	Page Number
**Administrative information**			
Title	#1	Descriptive title identifying the study design, population, interventions, and, if applicable, trial acronym	1

For peer review only

Trial registration	#2a	Trial identifier and registry name. If not yet registered, name of intended registry	3
Trial registration: data set	#2b	All items from the World Health Organization Trial Registration Data Set	
Protocol version	#3	Date and version identifier	4
Funding	#4	Sources and types of financial, material, and other support	2, 24
Roles and responsibilities: contributorship	#5a	Names, affiliations, and roles of protocol contributors	23
Roles and responsibilities: sponsor contact information	#5b	Name and contact information for the trial sponsor	24
Roles and responsibilities: sponsor and funder	#5c	Role of study sponsor and funders, if any, in study design; collection, management, analysis, and interpretation of data; writing of the report; and the decision to submit the report for publication, including whether they will have ultimate authority over any of these activities	9
Roles and responsibilities: committees	#5d	Composition, roles, and responsibilities of the coordinating centre, steering committee, endpoint adjudication committee, data management team, and	9 and 17-19

For peer review only

		other individuals or groups overseeing the trial, if applicable (see Item 21a for data monitoring committee)	
**Introduction**			
Background and rationale	#6a	Description of research question and justification for undertaking the trial, including summary of relevant studies (published and unpublished) examining benefits and harms for each intervention	5-6
Background and rationale: choice of comparators	#6b	Explanation for choice of comparators	15-16
Objectives	#7	Specific objectives or hypotheses	6
Trial design	#8	Description of trial design including type of trial (eg, parallel group, crossover, factorial, single group), allocation ratio, and framework (eg, superiority, equivalence, non-inferiority, exploratory)	6-7
**Methods: Participants, interventions, and outcomes**			
Study setting	#9	Description of study settings (eg, community clinic, academic hospital) and list of countries where data will be collected. Reference to where list of study sites can be obtained	9

For peer review only

Eligibility criteria	#10	Inclusion and exclusion criteria for participants. If applicable, eligibility criteria for study centres and individuals who will perform the interventions (eg, surgeons, psychotherapists)	7-8
Interventions: description	#11a	Interventions for each group with sufficient detail to allow replication, including how and when they will be administered	15-17
Interventions: modifications	#11b	Criteria for discontinuing or modifying allocated interventions for a given trial participant (eg, drug dose change in response to harms, participant request, or improving / worsening disease)	14, 21-22
Interventions: adherance	#11c	Strategies to improve adherence to intervention protocols, and any procedures for monitoring adherence (eg, drug tablet return; laboratory tests)	14-17
Interventions: concomitant care	#11d	Relevant concomitant care and interventions that are permitted or prohibited during the trial	15-17
Outcomes	#12	Primary, secondary, and other outcomes, including the specific measurement variable (eg, systolic blood pressure), analysis metric (eg, change from baseline, final value, time to event), method of aggregation (eg, median, proportion), and time point for each outcome. Explanation of the clinical relevance of chosen efficacy and harm outcomes is strongly recommended	10-13

For peer review only

Participant timeline	#13	Time schedule of enrolment, interventions (including any run-ins and washouts), assessments, and visits for participants. A schematic diagram is highly recommended (see Figure)	Table 1, Figure 1
Sample size	#14	Estimated number of participants needed to achieve study objectives and how it was determined, including clinical and statistical assumptions supporting any sample size calculations	9-10
Recruitment	#15	Strategies for achieving adequate participant enrolment to reach target sample size	9, 13-15
**Methods: Assignment of interventions (for controlled trials)**			
Allocation: sequence generation	#16a	Method of generating the allocation sequence (eg, computer-generated random numbers), and list of any factors for stratification. To reduce predictability of a random sequence, details of any planned restriction (eg, blocking) should be provided in a separate document that is unavailable to those who enrol participants or assign interventions	17
Allocation concealment mechanism	#16b	Mechanism of implementing the allocation sequence (eg, central telephone; sequentially numbered, opaque,	17, 18

For peer review only

		sealed envelopes), describing any steps to conceal the sequence until interventions are assigned	
Allocation: implementation	#16c	Who will generate the allocation sequence, who will enrol participants, and who will assign participants to interventions	17
Blinding (masking)	#17a	Who will be blinded after assignment to interventions (eg, trial participants, care providers, outcome assessors, data analysts), and how	7, 16-18
Blinding (masking): emergency unblinding	#17b	If blinded, circumstances under which unblinding is permissible, and procedure for revealing a participant's allocated intervention during the trial	22
**Methods: Data collection, management, and analysis**			
Data collection plan	#18a	Plans for assessment and collection of outcome, baseline, and other trial data, including any related processes to promote data quality (eg, duplicate measurements, training of assessors) and a description of study instruments (eg, questionnaires, laboratory tests) along with their reliability and validity, if known. Reference to where data collection forms can be found, if not in the protocol	10-15, 17

For peer review only

Data collection plan: retention	#18b	Plans to promote participant retention and complete follow-up, including list of any outcome data to be collected for participants who discontinue or deviate from intervention protocols	9, 13-15
Data management	#19	Plans for data entry, coding, security, and storage, including any related processes to promote data quality (eg, double data entry; range checks for data values). Reference to where details of data management procedures can be found, if not in the protocol	19
Statistics: outcomes	#20a	Statistical methods for analysing primary and secondary outcomes. Reference to where other details of the statistical analysis plan can be found, if not in the protocol	19-21
Statistics: additional analyses	#20b	Methods for any additional analyses (eg, subgroup and adjusted analyses)	19-21
Statistics: analysis population and missing data	#20c	Definition of analysis population relating to protocol non-adherence (eg, as randomised analysis), and any statistical methods to handle missing data (eg, multiple imputation)	19-21

Methods: Monitoring

Data monitoring: formal committee	#21a	Composition of data monitoring committee (DMC); summary of its role and reporting structure; statement of whether it is independent from the sponsor and	24

For peer review only

		competing interests; and reference to where further details about its charter can be found, if not in the protocol. Alternatively, an explanation of why a DMC is not needed	
Data monitoring: interim analysis	#21b	Description of any interim analyses and stopping guidelines, including who will have access to these interim results and make the final decision to terminate the trial	24
Harms	#22	Plans for collecting, assessing, reporting, and managing solicited and spontaneously reported adverse events and other unintended effects of trial interventions or trial conduct	12, 14-15, 21-22
Auditing	#23	Frequency and procedures for auditing trial conduct, if any, and whether the process will be independent from investigators and the sponsor	24
**Ethics and dissemination**			
Research ethics approval	#24	Plans for seeking research ethics committee / institutional review board (REC / IRB) approval	23
Protocol amendments	#25	Plans for communicating important protocol modifications (eg, changes to eligibility criteria, outcomes, analyses) to relevant parties (eg, investigators, REC / IRBs, trial participants, trial registries, journals, regulators)	23

For peer review only

Consent or assent	#26a	Who will obtain informed consent or assent from potential trial participants or authorised surrogates, and how (see Item 32)	14
Consent or assent: ancillary studies	#26b	Additional consent provisions for collection and use of participant data and biological specimens in ancillary studies, if applicable	14
Confidentiality	#27	How personal information about potential and enrolled participants will be collected, shared, and maintained in order to protect confidentiality before, during, and after the trial	23
Declaration of interests	#28	Financial and other competing interests for principal investigators for the overall trial and each study site	24
Data access	#29	Statement of who will have access to the final trial dataset, and disclosure of contractual agreements that limit such access for investigators	23
Ancillary and post trial care	#30	Provisions, if any, for ancillary and post-trial care, and for compensation to those who suffer harm from trial participation	24
Dissemination policy: trial results	#31a	Plans for investigators and sponsor to communicate trial results to participants, healthcare professionals, the public, and other relevant groups (eg, via publication, reporting in results databases, or other data sharing arrangements), including any publication restrictions	24

For peer review only

Dissemination policy: authorship	#31b	Authorship eligibility guidelines and any intended use of professional writers	24
Dissemination policy: reproducible research	#31c	Plans, if any, for granting public access to the full protocol, participant-level dataset, and statistical code	24

Appendices

Informed consent materials	#32	Model consent form and other related documentation given to participants and authorised surrogates	-
Biological specimens	#33	Plans for collection, laboratory evaluation, and storage of biological specimens for genetic or molecular analysis in the current trial and for future use in ancillary studies, if applicable	12-13

None The SPIRIT checklist is distributed under the terms of the Creative Commons Attribution License CC-BY-ND 3.0. This checklist can be completed online using https://www.goodreports.org/, a tool made by the EQUATOR Network in collaboration with Penelope.ai

For peer review only

BMJ Open

Efficacy of Metamizole versus Ibuprofen and a Short Educational Intervention versus Standard Care in Acute and Subacute Low Back Pain: A Study Protocol of a Randomized, Multicenter, Factorial Trial (EMISI Trial)

Journal:	*BMJ Open*
Manuscript ID	bmjopen-2020-048531.R1
Article Type:	Protocol
Date Submitted by the Author:	01-Jul-2021
Complete List of Authors:	Wertli, Maria M; University of Bern, Department of General Internal Medicine<br>Flury, Julian S.; University of Bern, Department of General Internal Medicine<br>Streit, Sven; University of Bern, Institute of Primary Health Care (BIHAM)<br>Limacher, Andreas; Universitat Bern Institut fur Sozial- und Praventivmedizin<br>Schuler, Vanessa; University of Bern, Department of General Internal Medicine<br>Ferrante, Asha-Naima; University of Bern, Department of General Internal Medicine<br>Rimensberger, Caroline; University of Bern, Department of General Internal Medicine<br>Haschke, Manuel; University of Bern, Clinical Pharmacology and Toxicology
**Primary Subject Heading**:	General practice / Family practice
Secondary Subject Heading:	Evidence based practice, Emergency medicine
Keywords:	PAIN MANAGEMENT, Back pain < ORTHOPAEDIC & TRAUMA SURGERY, NEUROPHYSIOLOGY

SCHOLARONE™
Manuscripts

I, the Submitting Author has the right to grant and does grant on behalf of all authors of the Work (as defined in the below author licence), an exclusive licence and/or a non-exclusive licence for contributions from authors who are: i) UK Crown employees; ii) where BMJ has agreed a CC-BY licence shall apply, and/or iii) in accordance with the terms applicable for US Federal Government officers or employees acting as part of their official duties; on a worldwide, perpetual, irrevocable, royalty-free basis to BMJ Publishing Group Ltd ("BMJ") its licensees and where the relevant Journal is co-owned by BMJ to the co-owners of the Journal, to publish the Work in this journal and any other BMJ products and to exploit all rights, as set out in our licence.

The Submitting Author accepts and understands that any supply made under these terms is made by BMJ to the Submitting Author unless you are acting as an employee on behalf of your employer or a postgraduate student of an affiliated institution which is paying any applicable article publishing charge ("APC") for Open Access articles. Where the Submitting Author wishes to make the Work available on an Open Access basis (and intends to pay the relevant APC), the terms of reuse of such Open Access shall be governed by a Creative Commons licence – details of these licences and which Creative Commons licence will apply to this Work are set out in our licence referred to above.

Other than as permitted in any relevant BMJ Author's Self Archiving Policies, I confirm this Work has not been accepted for publication elsewhere, is not being considered for publication elsewhere and does not duplicate material already published. I confirm all authors consent to publication of this Work and authorise the granting of this licence.

For peer review only

Efficacy of Metamizole versus Ibuprofen and a Short Educational Intervention versus Standard Care in Acute and Subacute Low Back Pain: A Study Protocol of a Randomized, Multicenter, Factorial Trial (EMISI Trial)

Maria M. Wertli[1], Julian S. Flury[1], Sven Streit[2], Andreas Limacher[3], Vanessa Schuler[1], Asha-Naima Ferrante[1,4], Caroline Rimensberger[1], Manuel Haschke[5]

[1]Department of General Internal Medicine, Inselspital, Bern University Hospital, University of Bern, Bern, Switzerland

[2]Institute of Primary Health Care (BIHAM), University of Bern, Bern, Switzerland.

[3]CTU Bern, University of Bern, Bern, Switzerland

[4]Faculty of Human Sciences and Department of Psychology, University of Bern, Bern Switzerland

[5]Clinical Pharmacology and Toxicology, Department of General Internal Medicine, Inselspital, Bern University Hospital, University of Bern, Switzerland

Corresponding author

Maria Wertli, MD PhD
Department of General Internal Medicine
Bern University Hospital
Freiburgstrasse 8
3010 Bern
Mail: Maria.Wertli@insel.ch
Phone: +41 31 632 01 46

For peer review only

Abstract

INTRODUCTION Low back pain (LBP) is among the top three most common diseases worldwide, resulting in a life with pain-related disability. To date, no study has assessed the efficacy of metamizole (dipyrone), a non-opioid analgesic and antipyretic prodrug compared to the conventional NSAID ibuprofen, in patients with an acute low back pain episode. Further, it is unclear, whether a short-educational intervention is superior to usual care alone.

OBJECTIVES The objective of this study is to assess first, whether metamizole is non-inferior to ibuprofen in a new episode of acute or subacute LBP. Second, we aim to assess whether a short educational intervention including evidence-based patient information on the nature of LBP is superior to usual care alone.

METHODS AND ANALYSIS An investigator-initiated multicenter, randomized, double blind trial using a factorial design will be performed. A total of 120 participants with a new episode of LBP will be recruited from GP practices, outpatient clinics, and from emergency departments, and randomized into four different treatment groups: ibuprofen alone, ibuprofen and short-intervention, metamizole alone, metamizole and short-intervention. The primary endpoint for the medical treatment will be change in pain assessed on an 11-point numeric rating scale (NRS) after 14 days. The primary outcome for the short intervention will be change in the Core Outcome Index (COMI) assessed after 42 days.

ETHICS, DISSEMINATION, AND FUNDING This study has been approved by the responsible Ethics Board (Ethikkommission Bern / 2018-01986) and the Swiss Agency for Therapeutic Products (Swissmedic / 2019DR4002). Results will be published in open access policy peer-reviewed journals. The study is funded by the Swiss National Science Foundation (grant number 32003B_179346).

For peer review only - http://bmjopen.bmj.com/site/about/guidelines.xhtml

TRIAL REGISTRATION NCT04111315

For peer review only

For peer review only - http://bmjopen.bmj.com/site/about/guidelines.xhtml

Article Summary

Strengths and limitations of this study

- This is the first randomized double-blind study assessing the analgesic effect of metamizole compared to the conventional NSAID ibuprofen in patients with acute or subacute LBP presenting to the GP or emergency department.
- In addition, this study will assess whether a short educational intervention is more effective in LBP patients compared to usual care.
- Due to the high proportion of patients who will not require extended pain treatment during an acute LBP episode, the trial design mirrors current clinical practice.
- The primary endpoint pain intensity will be assessed after 14 days. The primary outcome for the short intervention will be assessed after 42 days.
- While patients, physicians, and researchers are blinded to the pharmacological treatment, blinding is not feasible for the short educational intervention.

Keywords

low back pain, randomized controlled trial, pain medication ibuprofen, NSAID, metamizole, psychological intervention, outcome

Word count:

5631 words.

Protocol Version 2, July 1. 2021

For peer review only - http://bmjopen.bmj.com/site/about/guidelines.xhtml

Introduction

Low back pain (LBP) is among the top three most common diseases worldwide, resulting in a life with pain-related disability.[1] In patients with persistent LBP over 3 months, the risk for chronic pain increases sharply[2] and effective interventions should aim to prevent pain persistence without overtreatment[3]. Therefore, treatment guidelines recommend the use of pain medication and to keep patients physically active during an acute LBP episode.[4 5] Despite the frequency of LBP, only few high-quality studies assessed the efficacy of pain medication. Systematic reviews of randomized clinical trials found opioids to be no more effective than non-opioid medication in LBP[6 7] but opioids were associated with potentially severe side-effects.[4 7 8] Therefore, non-opioid pain medications that are safe and effective are urgently needed to control pain.

Current treatment guidelines recommend non-steroidal anti-inflammatory drugs (NSAIDs) for acute pain control.[4 9] However, adverse events including kidney injury and gastrointestinal bleeding limit their use in many patients. Metamizole (dipyrone) is a non-opioid analgesic and antipyretic with a favorable gastrointestinal and renal safety profile.[10] For example, metamizole use was associated with a lower risk for gastrointestinal bleeding compared to NSAIDs and for hepatotoxicity compared to paracetamol.[11] Although Metamizole has been withdrawn from the market or never approved in many countries including the USA, Canada, and UK,[12] it is frequently used in many other countries such as Germany,[13] Spain,[12] Switzerland,[14] some Eastern European, and South American countries.[12 15] Despite its increasing use in many European countries,[14 16] the role of metamizole for the treatment of LBP is unclear and has so far not been systematically studied. This may be due to an ongoing controversy regarding the risk of metamizole-associated agranulocytosis.[12 15] Due to the rare occurrence, incidence rates have been based on case control studies and pharmacovigilance data. The incidence of the metamizole induced agranulocytosis was

For peer review only - http://bmjopen.bmj.com/site/about/guidelines.xhtml

estimated between 0.5 and 3.5 cases per 1 million metamizole users over one week per year.[17-28] Even though the overall risk of agranulocytosis is higher compared to other analgesics, it remains a rare event occurring only in a small proportion of susceptible patients[17-25 27-29] and the overall safety profile of metamizole is still favorable compared to NSAIDs or opioids.[30]

Many patients have reservations against regular intake of pain medication and, contrary to guideline recommendations, limit physical activity to keep pain manageable without medication.[31] Patient education encouraging physical activity may help to improve pain control and function. For example, a Cochrane review found that 2.5-hour educational sessions were more effective than no intervention on the return-to-work rate in patients with acute/subacute LBP.[32] Such time-intensive interventions may not be necessary in all patients and are not feasible in a busy primary care practice. Short educational interventions would be more practical, however, to date it is unclear whether they also have a positive effect on outcome in patients with a new LBP episode.

Objectives

The first objective of this study is to assess whether metamizole is non-inferior to ibuprofen in patients with a new episode of acute or subacute LBP. The second objective is to assess whether a short educational intervention, including evidence-based patient information on the nature of LBP, is superior to usual care alone. Thus, this randomized trial will provide new knowledge regarding the efficacy of non-opioid pain medications in LBP. In addition, if the short educational intervention proves to be beneficial compared to usual care, the trial will help to improve care for patients with a new LBP episode.

For peer review only - http://bmjopen.bmj.com/site/about/guidelines.xhtml

Methods and analysis

The methods reporting of this randomized trial will follow the recommendations of the SPIRIT statement.[33]

Design: Multicenter, randomized, double-blind trial using a factorial design

The EMISI study is a multicenter, randomized, double-blind trial using a factorial design to investigate whether metamizole is non-inferior to ibuprofen in patients with a new onset LBP episode. Patients, physicians, and researchers will be blinded to the allocation of the study medication. A non-inferiority design was chosen as metamizole seems to be equally effective as ibuprofen in clinical practice. Furthermore, the study will assess whether a short educational intervention including evidence-based patient information is superior to usual care. Blinding patients about the short intervention is not possible but blinding is upheld in GPs and researchers (double blind). The primary evaluation of outcomes (see Outcome measure for details) will be performed at day 14 (comparison of metamizole to ibuprofen) and at day 42 (comparison of educational short intervention to usual care).

Eligibility criteria

Subjects fulfilling all of the following inclusion criteria are eligible for the study:

- Age 18 years or older
- Seeking care for new onset of non-specific or specific LBP (pain duration of less than 12 weeks LBP prior to the baseline visit)
- The GP plans to prescribe a non-opioid pain medication for pain control
- Informed consent documented by signature

For peer review only - http://bmjopen.bmj.com/site/about/guidelines.xhtml

Exclusion criteria are:

- Chronic use of opioids
- Presence of red flags (serious neurological deficit requiring surgery, infection, vertebral fracture)
- Known intolerance to the study medication (i.e. previous acute allergic reaction to NSAIDs or metamizole)
- Active malignancy and / or history of a (previous) hematologic disorder (history of agranulocytosis)
- History of anemia (hemoglobin of <100.0 mg/L), neutropenia (leucocyte count of < 3.0G/L), thrombocytopenia (<100G/L)
- Known contraindications against the study medications: previous gastrointestinal ulcer/bleeding, inflammatory bowel disease, heart failure (NYHA III-IV), liver failure (liver cirrhosis, ascites), renal insufficiency (estimated glomerular filtration rate (eGFR) < 60 ml/min/1,73 m2) or previous acute kidney injury (AKI stage 2 according to the KDIGO definition)
- Immune deficiency or under immunosuppressant treatment
- Known or suspected non-compliance, drug or alcohol abuse
- Participation in another study within 30 days preceding randomization and during the present study or previous enrolment into the current study
- Enrolment of the investigator, his/her family members, employees, and other dependent persons
- Pregnancy: In women of childbearing age, a negative pregnancy test (urine or blood test as available in the primary care practice) before inclusion is required. Women who are not willing to use safe contraception (condom or birth control pill)

For peer review only - http://bmjopen.bmj.com/site/about/guidelines.xhtml

during the trial, intention to become pregnant during the trial, pregnancy, or breast feeding.

Study setting

The EMISI trial will be coordinated by the central study team at the Division of General Internal Medicine, University Hospital Bern, Switzerland. Patients will be recruited from outpatient clinics, from GP practices, and from emergency departments. The overall study duration of 42 days (6 weeks) was chosen because treatment guidelines recommend further assessment of patients for multidisciplinary treatment if LBP persists for more than 6 weeks despite pain medication and the advice to stay active. This is a investigator initiated trial. The funder will have no role in the study design, the trial oversight, data collection, analysis of the study, and publication of the results.

Estimated sample size and power

Both interventions are considered for determining the sample size for this factorial trial.[34] We expect the two primary hypotheses to be independent of each other and do not expect an interaction between both interventions. Therefore, we powered each hypothesis individually, but accounted for multiple testing by adjusting the type-I-error rate by setting the alpha level to 0.025 in each test to keep the overall type-I error rate at 0.05. Thus, we aimed to include 120 patients to account for a drop-out of 10% which will allow to proof non-inferiority of the metamizole treatment at a one-sided alpha-level of 2.5% with a power of 90%.

For peer review only - http://bmjopen.bmj.com/site/about/guidelines.xhtml

The sample size calculation for the comparison of metamizole vs. ibuprofen was based on a difference in the change of pain score in the numeric rating scale (NRS; range 0 to 10, higher score indicates more pain). We considered a change of ≥2 points between the two groups as clinically relevant and a change of ≤1 point as negligible.[35 36] Therefore, we set the non-inferiority margin to -1 score point. Based on a two-sample means test and a standard deviation of 1.6,[35] we will need 108 patients (54 per group) to proof non-inferiority of the metamizole treatment at a one-sided alpha-level of 2.5% with a power of 90%.

The sample size for the primary outcome comparison short intervention vs. usual care (superiority) is based on a change in Core Outcome Measures Index (COMI; range 0 to 10, higher score indicates higher level of complaint, see primary outcomes) from baseline to week 6. We hypothesize that there is a difference between the two intervention groups regarding COMI. Mannion et al.[37] reported a minimal clinically important difference for improvement in the COMI of 2.2 points and standard deviations for changes in COMI ranging from 1.7 to 2.5 points. We see a difference of 2.2 points between the two groups as clinically relevant and assume a standard deviation of 2.2 points. Based on a two-sample means test, we will need 54 patients (27 per group) to detect a difference in the change of the COMI at a two-sided alpha-level of 2.5% with a power of 90%.

Primary outcome

Table 1 provides an overview of measurements according to the Standard Protocol Items: Recommendations for Interventional Trials. The primary outcome for the comparison of metamizole vs. ibuprofen is the change in pain on the NRS (range 0-10 points) from baseline to follow-up at day 14 (non-inferiority). Pain intensity will be recorded as average pain intensity for the past 24 hours on an NRS scale from 0 to 10,

For peer review only - http://bmjopen.bmj.com/site/about/guidelines.xhtml

scored from 0 (no pain) to 10 (worst possible pain). The rational for assessing the effectiveness outcome at 14 days was based on the double-blind RCT comparing paracetamol to placebo.[38]

The primary outcome for the comparison of the short intervention vs. usual care is the change in the COMI sum-score (range 0-10) from baseline to 42 days follow-up (superiority). The COMI is a short, multidimensional, validated outcome instrument with excellent psychometric properties recommended for the monitoring of the outcome in patients undergoing treatment for LBP.[37] The COMI sum score is calculated as described in the validation paper.[39 40] The pain scales (item 2a and 2b) are scored 0-10, whilst the category scales (items 3-7) are scored as 0, 2.5, 5.0, 7.5, 10.0. The sum score (0-10) for the whole COMI scale is calculated based on the following 5 domains:

- Pain domain: higher of the two pain scores (higher of 2a and 2b) is taken as the score
- Disability domain: the average of the two disability items (6 and 7)
- Back related function (item 3)
- Symptom specific well-being (item 4)
- General quality of life (item 5)

The five domain scores are then averaged to give a COMI score from 0-10.

Two additional questions cover the satisfaction with treatment outcome. A minimal clinically important difference (MCID) found to be relevant for patients was a reduction in COMI score of ≥2.2 points.[37]

Secondary outcome

All outcome measures used in this study were recommended by the NIH Task force and other expert panels to study back pain.[41 42] **Table 1** provides an overview of

For peer review only - http://bmjopen.bmj.com/site/about/guidelines.xhtml

measurements according to the Standard Protocol Items: Recommendations for Interventional Trials.[33]

Secondary outcome measures include:

- Change in pain on the numeric rating scale (NRS, range 0-10 points) from baseline to follow-up at day 42
- Change in the Core Outcome Measures Index (COMI) sum-score (range 0-10) from baseline to 14 days follow-up
- The proportion of patients with a minimal clinically important difference (MCID) in the NRS (≥2 points) and COMI (≥2.2 points) at days 14 and 42
- Time to recovery (recovery defined as the first day with a NRS of <2 points sustained for 2 consecutive days)
- Change in overall pain intensity from baseline to follow-up at day 14 and day 42. Overall pain intensity: average of ratings for average pain, pain at rest, and pain during activity during the last 24 hours.
- Area under effect curve (AUEC day 0 to day 14) NRS
- Adverse events (AE)
- Time to stopping treatment
- Global effectiveness of the treatment (COMI question ‘‘how much did the operation/treatment help your back problem?’’ 5-point Likert scale from ‘‘helped a lot’’ to ‘‘made things worse’’)
- Daily physical activity (daily step count)
- Use of pain medication and additional rescue medication
- Additional treatment (physical therapy, injections)
- Return to work (yes / no)

For peer review only - http://bmjopen.bmj.com/site/about/guidelines.xhtml

- Treatment satisfaction (COMI)
- Quality of life as assessed by the 5-level version of the European Quality of Life-5 Dimensions questionnaire (EQ-5D)
- Psychological factors (STarT-Back Tool)[43]

Exploratory variables

Response to pain medications may be influenced by individual pharmacokinetic and pharmacodynamics factors. Therefore, we will collect blood samples during visit 2 (day 14) to assess the following factors that may influence the individual response to the pain medications. First, we will measure plasma concentrations of ibuprofen and the four main metamizole metabolites (4-MAA, 4-AA, 4-FA, 4-AAA). Pharmacodynamic effects of metamizole correlate primarily with concentrations of the 4-MAA metabolite,[44] thus direct quantification of 4-MAA concentrations will add important information for the interpretation of the observed effects.[45]

Second, as individual response to ibuprofen may be affected by genetic polymorphisms of CYP isoforms 2C8 and 2C9, the study population will be characterized in terms of cytochrome P450 (CYP) 2C8/9 genotypes (CYP2C8 *1 and *3 alleles, CYP2C9 *1, *2, and *3 alleles). In subjects with alleles conferring low CYP2C8 and / or CYP2C9 activity, reduced ibuprofen clearance of both enantiomers has been described, resulting in several-fold increased exposure and prolonged half-life compared to subjects with wild-type alleles.[46 47]

Study procedures

Study procedures are summarized in the study flow chart (**Figure 1**). Each participating site will receive a supply of sequentially coded, blinded, and sealed

For peer review only - http://bmjopen.bmj.com/site/about/guidelines.xhtml

intervention packs. All patients presenting with LBP will be screened for potential inclusion into the study. After obtaining informed consent by the physician, patient information will be entered in a web-based clinical data management system (CDMS). After patient registration in the CDMS and confirmation of all inclusion and exclusion criteria, patients are randomized using sequentially coded and blinded intervention packs, thus ensuring concealment of allocation. The number of the intervention pack will be recorded in the CDMS.

Patients will complete a set of self-reported questionnaires (see Table 1 for details) on demographics (age, sex, work, and civic status), previous history, current medications, pain related information (NRS, pain drawing), disability (COMI, STarT Back tool), quality of life (European Quality of Life-5 Dimensions questionnaire (EQ5-D-5L), the Fear Avoidance Questionnaire (FABQ),[48] self-efficacy (Schmerzspezifische Selbstwirksamkeit (FESS[49])). Physicians will complete a questionnaire on clinical findings, the clinical diagnosis, previous and newly prescribed treatments.

Upon completion of the baseline evaluation, patients may open the intervention pack and start treatment. The intervention pack will contain a pill bottle and an envelope with the assignment of the short vs no intervention, and a pain diary.

Between the first and the second visit, patients will record their pain before intake of study drug, the number of study drugs and additional pain medications, and their daily physical activity (quantified by daily step counts using a commercially available wearable step tracking device (Garmin vivofit 4®).

To mitigate the risk for AEs, we will collect blood samples after the randomization in all patients. In case of a newly detected renal insufficiency (defined as estimated glomerular filtration rate (eGFR) < 60 ml/min/1.73 m^2), leucopenia (leucocyte count <

For peer review only - http://bmjopen.bmj.com/site/about/guidelines.xhtml

3.0G/L), thrombocytopenia (defined as <100 G/L) and/or anemia ((hemoglobin <100g/L), we will stop the study medication.

During the second visit (day 14), patients will return the pill bottles and the pain diary to the GPs office where they are collected for pill count. The patient will receive a new drug pack and pain diary. A blood sample will be collected (creatinine, blood count, drug concentrations and genotyping), and self-reported questionnaires and a clinical assessment will be completed. The GP will record the clinical findings and additional pharmacological and non-pharmacological treatments.

To improve adherence, study staff will call patients between the first and the second visit and between the second and the final visit and ask for adverse events and additional pain treatment. As we expect a high proportion of patients to have recovered by day 28 (second phone call), researchers will then determine whether a clinical visit on day 42 is necessary. In case patients have recovered by day 28, patients do not need to attend the clinical visit. Patients will be asked whether they would be willing to participate in an in-person interview at the end of the study. The aim of the interview is to assess factors that were relevant for patients during their LBP treatment. The interview will be recorded (video and audio) for qualitative analysis using standard grounded theory method according to Philipp Mayring.[50]

A final assessment will be done on day 42 during a clinical visit in patients who had persisting symptoms on day 28. Patients will return the pain diary and the pill bottles with the remaining pills to the GPs office where they are collected for pill count. Data collection includes self-reported validated questionnaires. The GP will record the clinical findings and additional pharmacological and non-pharmacological treatments. A final blood sample (serum creatinine and blood count) will only be collected if patients are still taking study medication. Patients who do not need to attend the

For peer review only - http://bmjopen.bmj.com/site/about/guidelines.xhtml

clinical visit will receive a final identical set of questionnaires by mail and will be asked for AE/SAE and additional treatments during a phone call. Patients will return the questionnaires together with the pain diary and the pill bottles to the central study team. To assess the success of blinding, patients will be asked at the end of the study what treatment group they believe they had been assigned to (metamizole or ibuprofen).

Pharmacological intervention

During the first four days, we will compare a fixed-dose regimen of metamizole 500mg to 500 mg ibuprofen lysine (lysine-2-[4-isobutylphenyl]-propionate, corresponds to 292.6 mg ibuprofen acid) three times two capsules per day (**Table 2**). Ibuprofen was chosen as a comparator because it is a well-studied and the most frequently prescribed NSAID in Switzerland[14] with a similar dosing interval as metamizole. The initial dose of both medications is the currently used and recommended dose for the treatment of acute pain (common daily dose for metamizole 3000mg (maximum daily dose 4000mg) and 3000mg ibuprofen lysine (maximum daily dose 4000mg)). Ibuprofen lysine 3000mg corresponds to 1800mg ibuprofen acid. The ibuprofen lysine formulation was chosen to facilitate blinding of the study drug, as ibuprofen lysine 500mg was the only ibuprofen formulation available on the Swiss market, which could be prescribed with identical number of capsules and at identical intervals as metamizole. The lysine salt of ibuprofen shows better solubility and faster absorption compared with the acid form.[44 51] However, overall extent of absorption and analgesic effect in an acute pain model did not differ from the acid form.[52]

In LBP, current clinical practice is to prescribe an initial fixed dose regimen followed by an as-needed regimen that allows patients to adjust the daily dose depending on their pain intensity. To mirror clinical practice, patients will be allowed after the first four days

For peer review only - http://bmjopen.bmj.com/site/about/guidelines.xhtml

to decrease the intake of the study medication according to a dose reduction schedule (as needed phase). From day 4 until recovery patients may reduce the daily pain medication intake once their pain has decreased to less than 4 points on the NRS (range 0-10 points) every two days. The treatment duration will thus depend on the duration of pain. Patients are considered to have recovered when their pain is below 2 (NRS 0-10) on 2 consecutive days after they have stopped intake of pain medication (end of treatment).

Non-pharmacological educational short intervention

Patients assigned to the short intervention will receive together with the pain medication a short leaflet focusing on the nature of non-specific LBP and the importance of staying active. A second leaflet contains exercises to alleviate LBP. The content of the short information leaflet is based on the *The Back Book*.[53] Further, all patients assigned to the short intervention will receive a 10-minute standardized telephone intervention delivered by a trained member of the study team during the first 4 treatment days to help patients understand the non-dangerous nature of LBP, and to explain the purpose of pain medication to facilitate movement. In addition, patients will be encouraged to stay active and to perform the exercises as proposed in the exercise leaflet.

Concomitant interventions

In a double-blind RCT comparing paracetamol to placebo for LBP in the primary care setting, rescue medications were infrequently used.[38] Therefore, physicians will be allowed to prescribe additional rescue medications recommended by treatment guidelines at their discretion. Additional medication use is recorded in the pain diary, during the phone calls, and the clinical visits. Further, information on any use of physical therapy, massage or other treatments is collected during the follow-up visits at days 14

For peer review only - http://bmjopen.bmj.com/site/about/guidelines.xhtml

and 42. Although GPs will be instructed to withhold physical therapy until day 14 (i.e. the primary effectiveness endpoint for metamizole vs. ibuprofen) if the clinical situation allows, additional care including physical therapy and relaxation strategies recommended by guidelines will be at the discretion of the treating GP. GPs will record any additional prescription of addiational care. Concomitant treatments will be treated as co-variates to assess their potential impact on overall efficacy (performance bias).

Randomization and allocation procedures

The allocation sequence (randomization list) will be generated by an independent data manager at the CTU Bern who is not otherwise involved in the trial. Randomization will be blocked and stratified according to study sites. To ensure concealment of allocation, block size will not be communicated. After patient data is registered in the CDMS, eligibility is confirmed and signed informed consent is obtained, patients will be assigned to the next sequentially coded intervention pack that is available at the recruiting site. Patients are randomized 1:1:1:1 into one of four groups: Metamizole + educational intervention vs. metamizole + standard care vs. ibuprofen + educational intervention vs. ibuprofen + standard care. The intervention packs will contain a pill bottle and an envelope with the assignment of the short vs. no intervention. The intervention packs will be prepared according to the randomization list by an independent hospital pharmacist not involved in the study.

Blinding

Staff at the hospital pharmacy not involved in patient recruitment or other trial activities will fill the gelatin capsules with the study medication and label the pill bottles according to a standard operating procedure and based on the randomization list provided by the CTU Bern. The study medication will be delivered in identically labeled sealed pill bottles

For peer review only - http://bmjopen.bmj.com/site/about/guidelines.xhtml

containing capsules of identical appearance. The randomization list will be kept electronically at the CTU Bern in a password-protected file. Study personnel and physicians involved in patient recruitment, treatment, and outcome assessment will have no access to the list.

Due to the nature of the short educational intervention, no blinding of patients receiving an information leaflet and a telephone call is possible. Therefore, contamination and performance bias are important to consider. The envelope including the leaflet will be sealed and the patients will be instructed to open de envelope outside the GPs office. GPs will not be informed about the group assignment and the content and aim of the educational intervention. Patients assigned to the educational intervention will receive a telephone call according to a predefined script by a member of the study team otherwise not involved in patient recruitment, outcome assessment, and data analysis. To minimize the risk of detection bias, the study outcomes are collected in pain diaries and self-reported questionnaires (NRS at day 14, COMI at day 42). Study personnel that will contact patients by phone for reminders and to collect information on adverse events will be blinded to the group assignment. To assess differences in additional treatments (performance bias), patients and GPs will record additional treatments other than the study medication and the educational information (e.g. additional medication use, physical therapy, injections).

The statistician responsible for the final analysis will also have no access to the list until the primary analysis of the trial is finished. Data analysis will be done according to a prespecified analysis plan before the randomization code will be broken. Patients, physicians, and data analysts will be blinded with regard to the allocation to the study medication until after the primary analysis. To assess the success of blinding, we will ask patients at the end of the study into which group they belief they had been assigned to. Data on all recruited patients will be collected and regularly monitored.

For peer review only

Statistical analysis plan

The statistical analysis will be performed in compliance with the International Conference on Harmonisation's Good Clinical Practice guidelines and the report will be developed in line with the Consolidated Standards of Reporting Trials statement.

To assess the efficacy for the two primary outcomes, we will use two analysis sets (see **Figure 1**). The full analysis set (FAS) includes all randomized patients for the intention-to-treat (ITT) analysis, i.e. all patients will be analyzed in the group they were assigned to. The *per protocol (PP) set* is based on the FAS, excluding patients with major protocol violations, patients who were excluded due to the predefined stop criteria, patients withdrawing from the trial within 3 days of treatment start, patients undergoing surgery, and patients that did not receive the randomized treatment or intervention.

We will present patient and procedural characteristics using descriptive statistics (mean and standard deviation or median and interquartile range for continuous variables and frequency and percentage for categorical variables).

To compare the efficacy of metamizole and ibuprofen (non-inferiority), we use an ITT (FAS set) and a PP analysis (PP set) for the primary outcome (NRS change from baseline to follow-up at 14 days). Both analyses need to meet non-inferiority in order to claim success. The difference in the change in NRS between the two groups will be calculated from a linear model, adjusted for the baseline NRS value and the type of intervention (short intervention vs standard care). If the lower one-sided 97.5% confidence limit of the difference lies above the non-inferiority margin of -1, non-inferiority will be claimed. If we can demonstrate non-inferiority, we will additionally test for superiority at a two-sided significance level of 2.5%. Since testing is nested (i.e. hierarchical), this procedure has no implication on the overall type-I error rate.

For peer review only - http://bmjopen.bmj.com/site/about/guidelines.xhtml

To compare the primary outcome between the short educational intervention to usual care (superiority), we use ITT analysis (COMI change from baseline to day 42). The difference in the change in COMI between the two groups will be calculated and tested at a two-sided significance level of 2.5% using a linear model, adjusted for the baseline COMI and the type of drug treatment. Differences will be presented with a two-sided 97.5% confidence interval (CI) and the regular 95% CI.

We will assess both primary outcomes for interaction. We will introduce an interaction term between the treatment indicator and the intervention indicator to test whether in A) the effect of the metamizole treatment on the change in NRS at day 14 is different in patients that receive the short intervention compared with the standard care group and in B) whether the effect of the short intervention on the change in COMI at day 42 is different in patients that receive metamizole compared with patients that receive ibuprofen.

Secondary continuous outcomes will be analyzed as the primary outcomes. Besides the indicator for the drug treatment or intervention, models will be adjusted for the baseline value if available. Secondary binary outcomes will be analyzed using logistic regression models adjusted for the indicator for the drug treatment or intervention, as applicable. Time-to-event outcomes will be analyzed using Cox regression adjusted for the indicator for the drug treatment or intervention, as applicable. For all secondary effect measures, we will display regular two-sided 95% CI and accompanying p-values.

Further, we will perform additional PP analyses for the primary outcome of the comparison between the two intervention arms as well as for all secondary outcomes. If outcomes are missing, we will employ multiple imputation in the primary analysis and additionally perform an available case analysis disregarding missing data. Continuous outcomes that are measured several times during follow-up will additionally be evaluated

For peer review only - http://bmjopen.bmj.com/site/about/guidelines.xhtml

in a repeated measures mixed-effects linear model, additionally introducing a random intercept for patients into the model.

Exploratory analysis will be conducted with regards to predictors for treatment responses. We will explore the association between clinical predictors (e.g. depression, fear avoidance beliefs, social factors), drug levels, or genetic mutations and the treatment response in linear models. We will perform subgroup analyses according to gender, genetic phenotypes, and drug levels.

Safety outcomes will be reported as proportion and 95% CI separately for the two drug treatment groups using the safety population (i.e. all patients that have received at least one dose of the study drug and at least one safety-related visit or observation) set.

Patients with missing outcome data will be imputed using multiple imputation.

Safety

Protocol violations should not lead to treatment discontinuation unless they indicate a significant risk to patient safety.

Patients may be withdrawn from the study treatment for the following reasons:

- Hematologic disorders including leucopenia (defined as leucocyte count < 3.0G/L; thrombocytopenia (defined as <100 G/L), and anemia (decrease in hemoglobin ≥20g/L or <100g/L)
- Potential evidence for a hematologic disease (pathological blood smear needing further evaluation)
- Decreased kidney function (defined as an estimated creatinine clearance using the MDRD formula of < 50 ml/min/1.73m^2) or acute kidney injury stage 1 or higher

For peer review only - http://bmjopen.bmj.com/site/about/guidelines.xhtml

(defined according to the KDIGO definition: ≥1.5 times creatinine increase compared to baseline value or ≥26.5 micro mol/L creatinine increase)

- Development of toxicity which, in the investigators or GPs judgment, precludes further use of one of the study drugs
- Patients lost to follow-up or non-compliance

We will comply with all regulations concerning safety measures in clinical trials as set forth by the Swiss Agency for Therapeutic Products (Swissmedic). The investigators will report any serious adverse events occurring during clinical trials, independent of direct causal relationship with the treatment, within 24 hours. In case of a suspected unexpected serious adverse reaction (SUSAR), Swissmedic will be notified by the sponsor-investigator within the legal timelines. Unblinding will be permissible in case the information is required to ensure the patients safety in case of an AE.

All adverse events (AE) are reviewed by a safety committee and graded according to the National Health Service (NIH) Common Terminology Criteria for Adverse Events (CTCAE v5.0).[54]

Patient and public involvement

In this study, we use information material developed by patient advocacy groups. The results of this study will be communicated to the patients and physicians participating in the trial. The results will also be communicated to patients' advocacy groups and may help to improve the information material. Further, we will assess the impact of the study on patients and factors that may be facilitators or barriers to improve pain and pain management in LBP during in person interviews.

For peer review only - http://bmjopen.bmj.com/site/about/guidelines.xhtml

Clinical implications

During an LBP episode treatment guideline recommend to alleviate pain using pain medication and to stay physically active. To alleviate pain, NSAIDs are frequently used. However, the use of NSAIDs may be limited because of common adverse effects. Metamizole has a favorable adverse effects profile and may be used as an alternative. However, there is a lack of data on its effectiveness and safety for the treatment of LBP. This is the first study that systematically investigates the efficacy of metamizole in LBP patients undergoing treatment over the course of several weeks. Therefore, this study will provide evidence to support or discourage the use of metamizole in LBP. The findings of this study will be especially relevant for those patients where NSAIDs should not be used. Moreover, this trial is expected to provide evidence for a highly needed non-opioid treatment alternative in musculoskeletal pain. In particular, because the use of opioids has been shown to be no more effective than non-opioid medication[55] and is associated with potentially severe adverse events.

Non-pharmacological educational measures may potentiate the effect of the pain medication in acute LBP by addressing misbeliefs and avoiding disuse. Despite good evidence that staying active during an acute LBP episode is more effective than bed rest,[56] more than 50% of the Swiss population report avoiding movements and increasing rest during a LBP episode.[57] The interaction between patient and doctor early on influences the recovery of patients and many patients may benefit from a short educational intervention that is easy to be implemented even in a busy GP practice in addition to pain medication. This is the first study that investigates the impact of a short educational intervention in addition to pain medication on the recovery and medication adherence in patients with acute and subacute LBP. Even a small individual effect may have a large impact on the overall societal burden of LBP. Our work will inspire novel approaches on how to improve adherence to medication and behavioral changes.

Ethics and dissemination

The trial will be performed in compliance with the Declaration of Helsinki and its amendments, and has been approved by the responsible Ethics Board (Ethikkommission Bern/ 2018-01986) and the Swiss Agency for Therapeutic Products (Swissmedic / 2019DR4002). Important protocol modifications will be communicated to

For peer review only - http://bmjopen.bmj.com/site/about/guidelines.xhtml

the Ethics Board and participating study sites. Potential participants must provide written informed consent before entering the study. Subjects can leave the study at any time for any reason without any negative consequences for their further treatment. When a patient ends the treatment phase of the study prematurely, we will record the date and reason for early treatment discontinuation. If possible, the end of treatment evaluations will be collected before the patient is started on any other therapeutic intervention. Insurance coverage will be provided for all study participants by the study sponsor.

The results of the main trial and each of the secondary outcomes will be submitted for publication in open access peer-reviewed journals. Results will be disseminated to all participating centers and participants expressing interest. The EMISI study group is an independent academic research group, which will not employ professional writers. The EMISI study group will comply with the open access regulation of the Swiss National Science Foundation.

Authors' contributions

MMW, SS, AL, JF, and MH designed the study. AF, VS and CR critically commented on the methods and contributed to the development of the study. MMW and JF wrote the first draft of the manuscript. All authors revised the manuscript and approved the final version.

Funding statement

Efficacy of Metamizole versus Ibuprofen and a Short Educational Intervention versus Standard Care in Acute and Subacute Low Back Pain: A Randomized, Factorial Trial (The EMISI Trial) is supported by the Swiss National Science Foundation (grant number:

For peer review only - http://bmjopen.bmj.com/site/about/guidelines.xhtml

32003B_179346 / 1). This research project received no further support from any other funding agency.

Competing interests

Drs. Haschke and Wertli received a grant from the Swiss Science National Foundation for the study: grant number 32003B_179346. The funder plays no role in the study design, collection of data, analysis, and writing of the manuscript. There is no conflict of interest regarding intellectual content and proprietary affairs.

Data monitoring committee

Data monitoring will be conducted by the CTU Bern. Data collection, handling of the data, and the analysis will be done by the researchers at the University of Bern. The funder plays no role in the data acquisition, data storage, and analysis.

There is no interim analysis planned. Auditing will be performed by an independent auditor of the CTU at the central study site.

Trial sponsor investigator

Maria Wertli, MD PhD
Department of General Internal Medicine
Bern University Hospital
Freiburgstrasse 8
3010 Bern
Mail: Maria.Wertli@insel.ch
Phone: +41 31 632 01 46

For peer review only - http://bmjopen.bmj.com/site/about/guidelines.xhtml

Bibliography

1. Vos T, Allen C, Arora M, et al. Global, regional, and national incidence, prevalence, and years lived with disability for 310 diseases and injuries, 1990-2015: a systematic analysis for the Global Burden of Disease Study 2015. Lancet (London, England) 2016;**388**(10053):1545-602.
2. da C Menezes Costa L, Maher CG, Hancock MJ, et al. The prognosis of acute and persistent low-back pain: a meta-analysis. CMAJ: Canadian Medical Association journal 2012;**184**(11):E613-E24.
3. Itz CJ, Geurts JW, van Kleef M, et al. Clinical course of non-specific low back pain: a systematic review of prospective cohort studies set in primary care. European journal of pain (London, England) 2013;**17**(1):5-15.
4. Qaseem A, Wilt TJ, McLean RM, et al. Noninvasive treatments for acute, subacute, and chronic low back pain: A clinical practice guideline from the american college of physicians. Annals of internal medicine 2017;**166**(7):514-30.
5. van Tulder M, Becker A, Bekkering T, et al. Chapter 3. European guidelines for the management of acute nonspecific low back pain in primary care. European spine journal : official publication of the European Spine Society, the European Spinal Deformity Society, and the European Section of the Cervical Spine Research Society 2006;**15 Suppl 2**:S169-91.
6. Chou R, Deyo R, Friedly J, et al. Systemic Pharmacologic Therapies for Low Back Pain: A Systematic Review for an American College of Physicians Clinical Practice Guideline. Annals of internal medicine 2017;**166**(7):480-92.
7. Chaparro LE, Furlan AD, Deshpande A, et al. Opioids compared with placebo or other treatments for chronic low back pain: an update of the Cochrane Review. Spine 2014;**39**(7):556-63.
8. Hoffman EM, Watson JC, St Sauver J, et al. Association of Long-term Opioid Therapy With Functional Status, Adverse Outcomes, and Mortality Among Patients With Polyneuropathy. JAMA Neurol 2017;**74**(7):773-79.
9. Wong JJ, Cote P, Sutton DA, et al. Clinical practice guidelines for the noninvasive management of low back pain: A systematic review by the Ontario Protocol for Traffic Injury Management (OPTIMa) Collaboration. European journal of pain (London, England) 2017;**21**(2):201-16.
10. Konijnenbelt-Peters J, van der Heijden C, Ekhart C, et al. Metamizole (Dipyrone) as an Alternative Agent in Postoperative Analgesia in Patients with Contraindications for Nonsteroidal Anti-Inflammatory Drugs. Pain practice : the official journal of World Institute of Pain 2017;**17**(3):402-08.
11. Andersohn F, Konzen C, Garbe E. Systematic review: agranulocytosis induced by nonchemotherapy drugs. Annals of internal medicine 2007;**146**(9):657-65.
12. Stammschulte T, Ludwig WD, Muhlbauer B, et al. Metamizole (dipyrone)-associated agranulocytosis. An analysis of German spontaneous reports 1990-2012. European journal of clinical pharmacology 2015;**71**(9):1129-38.
13. Hoffmann F, Meinecke P, Freitag MH, et al. Who gets dipyrone (metamizole) in Germany? Prescribing by age, sex and region. Journal of clinical pharmacy and therapeutics 2015;**40**(3):285-8.
14. Wertli MM, Reich O, Signorell A, et al. Changes over time in prescription practices of pain medications in Switzerland between 2006 and 2013: an analysis of insurance claims. BMC health services research 2017;**17**(1):167.
15. Hoffmann F, Bantel C, Jobski K. Agranulocytosis attributed to metamizole: An analysis of spontaneous reports in EudraVigilance 1985-2017. Basic & Clinical Pharmacology & Toxicology 2020;**126**(2):116-25.

For peer review only - http://bmjopen.bmj.com/site/about/guidelines.xhtml

16. Hoffmann F, Schmiemann G. Pain medication in German nursing homes: a whole lot of metamizole. Pharmacoepidemiology and drug safety 2016;**25**(6):646-51.
17. Kramer M, Lane D, Hutchinson T. Risks of agranulocytosis and aplastic anemia. A first report of their relation to drug use with special reference to analgesics. The International Agranulocytosis and Aplastic Anemia Study. Jama 1986;**256**(13):1749-57.
18. Huber M, Andersohn F, Sarganas G, et al. Metamizole-induced agranulocytosis revisited: results from the prospective Berlin Case-Control Surveillance Study. European journal of clinical pharmacology 2015;**71**(2):219-27.
19. Ibanez L, Vidal X, Ballarin E, et al. Population-based drug-induced agranulocytosis. Archives of internal medicine 2005;**165**(8):869-74.
20. Basak GW, Drozd-Sokolowska J, Wiktor-Jedrzejczak W. Update on the incidence of metamizole sodium-induced blood dyscrasias in Poland. The Journal of international medical research 2010;**38**(4):1374-80.
21. Maj S, Centkowski P. A prospective study of the incidence of agranulocytosis and aplastic anemia associated with the oral use of metamizole sodium in Poland. Medical science monitor : international medical journal of experimental and clinical research 2004;**10**(9):Pi93-5.
22. Ibanez L, Vidal X, Ballarin E, et al. Agranulocytosis associated with dipyrone (metamizol). European journal of clinical pharmacology 2005;**60**(11):821-9.
23. Shapiro S, Issaragrisil S, Kaufman DW, et al. Agranulocytosis in Bangkok, Thailand: a predominantly drug-induced disease with an unusually low incidence. Aplastic Anemia Study Group. The American journal of tropical medicine and hygiene 1999;**60**(4):573-7.
24. van der Klauw MM, Goudsmit R, Halie MR, et al. A population-based case-cohort study of drug-associated agranulocytosis. Archives of internal medicine 1999;**159**(4):369-74.
25. Hamerschlak N, Maluf E, Biasi Cavalcanti A, et al. Incidence and risk factors for agranulocytosis in Latin American countries--the Latin Study: a multicenter study. European journal of clinical pharmacology 2008;**64**(9):921-9.
26. Hamerschlak N, Cavalcanti AB. Neutropenia, agranulocytosis and dipyrone. Sao Paulo medical journal = Revista paulista de medicina 2005;**123**(5):247-9.
27. Maj S, Lis Y. The incidence of metamizole sodium-induced agranulocytosis in Poland. The Journal of international medical research 2002;**30**(5):488-95.
28. Varonos DD, Santamouris S, Karambali S. The incidence of dipyrone-induced agranulocytosis in Greece during 1975. The Journal of international medical research 1979;**7**(6):564-8.
29. Blaser LS, Tramonti A, Egger P, et al. Hematological safety of metamizole: retrospective analysis of WHO and Swiss spontaneous safety reports. European journal of clinical pharmacology 2015;**71**(2):209-17.
30. Kötter T, da Costa BR, Fässler M, et al. Metamizole-Associated Adverse Events: A Systematic Review and Meta-Analysis. PloS one 2015;**10**(4):e0122918.
31. Rosser BA, McCracken LM, Velleman SC, et al. Concerns about medication and medication adherence in patients with chronic pain recruited from general practice. Pain 2011;**152**(5):1201-5.
32. Engers A, Jellema P, Wensing M, et al. Individual patient education for low back pain. The Cochrane database of systematic reviews 2008(1):Cd004057.
33. Chan AW, Tetzlaff JM, Gøtzsche PC, et al. SPIRIT 2013 explanation and elaboration: guidance for protocols of clinical trials. BMJ (Clinical research ed) 2013;**346**:e7586.
34. Chow SC, Shao J, Wang H. *Sample Size Calculations in Clinical Research.* 2nd ed. New York: Chapman & Hall/CRC, 2008.

For peer review only - http://bmjopen.bmj.com/site/about/guidelines.xhtml

35. Farrar JT, Young JP, Jr., LaMoreaux L, et al. Clinical importance of changes in chronic pain intensity measured on an 11-point numerical pain rating scale. Pain 2001;**94**(2):149-58.
36. Ostelo RW, Deyo RA, Stratford P, et al. Interpreting change scores for pain and functional status in low back pain: towards international consensus regarding minimal important change. Spine 2008;**33**(1):90-4.
37. Mannion AF, Porchet F, Kleinstuck FS, et al. The quality of spine surgery from the patient's perspective: part 2. Minimal clinically important difference for improvement and deterioration as measured with the Core Outcome Measures Index. European spine journal : official publication of the European Spine Society, the European Spinal Deformity Society, and the European Section of the Cervical Spine Research Society 2009;**18 Suppl 3**:374-9.
38. Williams CM, Maher CG, Latimer J, et al. Efficacy of paracetamol for acute low-back pain: a double-blind, randomised controlled trial. Lancet (London, England) 2014;**384**(9954):1586-96.
39. Mannion AF, Elfering A, Staerkle R, et al. Outcome assessment in low back pain: how low can you go? European spine journal : official publication of the European Spine Society, the European Spinal Deformity Society, and the European Section of the Cervical Spine Research Society 2005;**14**(10):1014-26.
40. Pochon L, Kleinstuck FS, Porchet F, et al. Influence of gender on patient-oriented outcomes in spine surgery. European spine journal : official publication of the European Spine Society, the European Spinal Deformity Society, and the European Section of the Cervical Spine Research Society 2016;**25**(1):235-46.
41. Deyo RA, Dworkin SF, Amtmann D, et al. Report of the NIH Task Force on research standards for chronic low back pain. Pain medicine (Malden, Mass) 2014;**15**(8):1249-67.
42. Pincus T, Santos R, Breen A, et al. A review and proposal for a core set of factors for prospective cohorts in low back pain: a consensus statement. Arthritis Rheum 2008;**59**(1):14-24.
43. Karstens S, Krug K, Hill JC, et al. Validation of the German version of the STarT-Back Tool (STarT-G): a cohort study with patients from primary care practices. BMC musculoskeletal disorders 2015;**16**:346.
44. Weiser T, Schepers C, Mück T, et al. Pharmacokinetic Properties of Ibuprofen (IBU) From the Fixed-Dose Combination IBU/Caffeine (400/100 mg; FDC) in Comparison With 400 mg IBU as Acid or Lysinate Under Fasted and Fed Conditions—Data From 2 Single-Center, Single-Dose, Randomized Crossover Studies in Healthy Volunteers. Clinical Pharmacology in Drug Development 2019;**8**(6):742-53.
45. Pierre SC, Schmidt R, Brenneis C, et al. Inhibition of cyclooxygenases by dipyrone. British journal of pharmacology 2007;**151**(4):494-503.
46. García-Martín E, Martínez C, Tabarés B, et al. Interindividual variability in ibuprofen pharmacokinetics is related to interaction of cytochrome P450 2C8 and 2C9 amino acid polymorphisms. Clinical Pharmacology & Therapeutics 2004;**76**(2):119-27.
47. Kirchheiner J, Meineke I, Freytag G, et al. Enantiospecific effects of cytochrome P450 2C9 amino acid variants on ibuprofen pharmacokinetics and on the inhibition of cyclooxygenases 1 and 2. Clinical Pharmacology & Therapeutics 2002;**72**(1):62-75.
48. Waddell G, Newton M, Henderson I, et al. A Fear-Avoidance Beliefs Questionnaire (FABQ) and the role of fear-avoidance beliefs in chronic low back pain and disability. Pain 1993;**52**(2):157-68.

For peer review only

For peer review only - http://bmjopen.bmj.com/site/about/guidelines.xhtml

49. Mangels M, Schwarz S, Sohr G, et al. Der Fragebogen zur Erfassung der schmerzspezifischen Selbstwirksamkeit (FESS) D Miaganogsetlisc ea, 2009;**55**(2):84-93.
50. Mayring P. *Qualitative Inhaltsanalyse. Grundlagen und Techniken*. 7. ed. Weinheim: Deutscher Studien Verlag, 2000.
51. Klueglich M, Ring A, Scheuerer S, et al. Ibuprofen extrudate, a novel, rapidly dissolving ibuprofen formulation: relative bioavailability compared to ibuprofen lysinate and regular ibuprofen, and food effect on all formulations. Journal of clinical pharmacology 2005;**45**(9):1055-61.
52. Kyselovič J, Koscova E, Lampert A, et al. A Randomized, Double-Blind, Placebo-Controlled Trial of Ibuprofen Lysinate in Comparison to Ibuprofen Acid for Acute Postoperative Dental Pain. Pain and Therapy 2020;**9**(1):249-59.
53. Burton AK, Waddell G, Tillotson KM, et al. Information and advice to patients with back pain can have a positive effect. A randomized controlled trial of a novel educational booklet in primary care. Spine 1999;**24**(23):2484-91.
54. U.S. Department of health and human services. Common Terminology Criteria for Adverse Events (CTCAE). In: U.S.Department of health and human services NIoH, national Cancer Institute, ed. June 14, 2010 ed: U.S.Department of health and human services,, 2009.
55. Friedman BW, Dym AA, Davitt M, et al. Naproxen With Cyclobenzaprine, Oxycodone/Acetaminophen, or Placebo for Treating Acute Low Back Pain: A Randomized Clinical Trial. Jama 2015;**314**(15):1572-80.
56. Hagen KB, Hilde G, Jamtvedt G, et al. Bed rest for acute low-back pain and sciatica. The Cochrane database of systematic reviews 2004(4):Cd001254.
57. Gerfin A. Rückenreport Schweiz 2011: Die Erhebung zur Rückengesundheit von Herrn und Frau Schweizer. 2011 August 30, 2011. https://www.rheumaliga.ch/assets/doc/CH_Dokumente/medienmitteilungen/facts-and-figures/Rueckenreport-Schweiz-2011_Rheumaliga-Schweiz.pdf.

For peer review only

Figure Title and Legend

Figure 1. Study flow chart

For peer review only

For peer review only - http://bmjopen.bmj.com/site/about/guidelines.xhtml

Tables

Table 1: Summary of study procedures based on Standard Protocol Items: Recommendations for Interventional Trials (SPIRIT) schedule

	Enrollment	Allocation	Short intervention	Phone call	Clinical visit	Phone call	Clinical visit or phone call
**Time point (days)**	-5 to 0	0	2	7	14	28	42
Clinical visit	1	1			2		**3 or ✆[4]**
Phone call			✆[2]	✆[3]		✆[3]	
Patient information & informed consent	x						
In-/Exclusion	x						
Demographics	x						
Medical History	x						
Physical examination	x						
Vital signs	x				x		(x)
Blood analysis	x				x		(x)
Pregnancy test	x						
Randomization		x					
Hand out study medication		x					
Daily intake of study medication		x					
Group 1 + 3: study medication only		x		x	x	x	x
Group 2 + 4: study medication + short intervention		x	x	x	x	x	x
Pain diary daily including:<br>- Pain<br>- additional treatments<br>- AE<br>- Daily physical activity		x	x	x	x	x	x
Adherence reminder[3]				x		x	

For peer review only - http://bmjopen.bmj.com/site/about/guidelines.xhtml

Screening for: [9]<br>- AE and SAE<br>- interrupting or discontinuing treatment				x	x	x	x
Collection of pill bottles for pill-count					x		x
Self-reported measures collected during visits							
Pain questionnaire (NRS)		x			x		x
COMI		x			x		x
STarT Back tool		x			x		x
Quality of life (EQ5-D-5L)		x			x		x
Fear avoidance (FABQ)		x					
Self-efficacy (FESS)		x					
Blinding control							x
Voluntary in-person interview on patient perspective							x

Legend: AE, adverse event; SAE, serious adverse event; NRS, numeric rating scale; COMI, Core Outcome Measures Index; EQ5-D-5L, European Quality of Life-5 Dimensions questionnaire; FABQ, Fear Avoidance Questionnaire, FESS, self-efficacy (Schmerzspezifische Selbstwirksamkeit)

For peer review only

For peer review only - http://bmjopen.bmj.com/site/about/guidelines.xhtml

Table 2: Oral doses for study medication – fixed dose and as-needed regimen

Group	Study medication	Fixed dose regimen (days 0 - 3)	As-needed regimen (after day 3): recommended reduction of the oral intake when LBP decreases
1	Ibuprofen only	500mg§ 2-2-2	2-2-2 (pain unchanged), 1-1-1 (decreased pain; NRS <4 points), 1-0-1, 1-0-0
2	Ibuprofen + short intervention	500mg§ 2-2-2	2-2-2 (pain unchanged), 1-1-1 (decreased pain; NRS <4 points), 1-0-1, 1-0-0
3	Metamizole only	500mg 2-2-2	2-2-2 (pain unchanged), 1-1-1 (decreased pain; NRS <4 points), 1-0-1, 1-0-0
4	Metamizole + short intervention	500mg 2-2-2	2-2-2 (pain unchanged), 1-1-1 (decreased pain; NRS <4 points), 1-0-1, 1-0-0

§500 mg ibuprofenum lysinum (lysine-2-[4-isobutylphenyl]-propionate) corresponds to 292.6 mg ibuprofen acid.

For peer review only - http://bmjopen.bmj.com/site/about/guidelines.xhtml

Abbreviations

AE	Adverse Event
CDMS	Clinical Data Management System
COMI	Core Outcome Measures Index
CRF	Case Report Form
EMISI	Efficacy of Metamizole versus Ibuprofen and a Short Educational Intervention versus Standard Care
GP	General Physician
ITT	Intention To Treat
LBP	Low Back Pain
NRS	Numeric Rating Scale
NSAIDs	Non-Steroidal Anti-Inflammatory Drugs

For peer review only

For peer review only - http://bmjopen.bmj.com/site/about/guidelines.xhtml

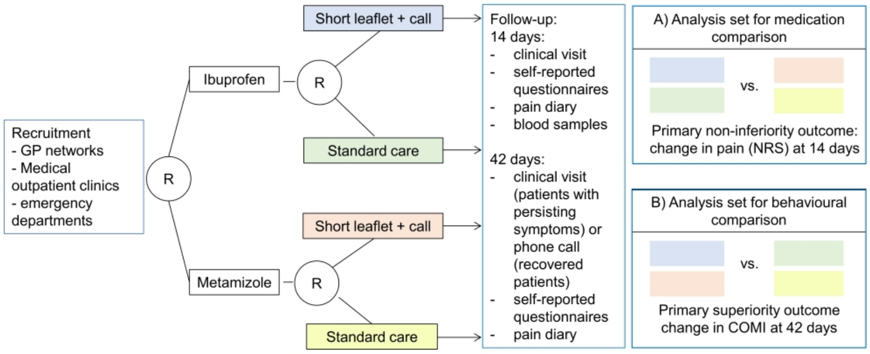

338x190mm (600 x 600 DPI)

Reporting checklist for protocol of a clinical trial.

Based on the SPIRIT guidelines.

Instructions to authors

Complete this checklist by entering the page numbers from your manuscript where readers will find each of the items listed below.

Your article may not currently address all the items on the checklist. Please modify your text to include the missing information. If you are certain that an item does not apply, please write "n/a" and provide a short explanation.

Upload your completed checklist as an extra file when you submit to a journal.

In your methods section, say that you used the SPIRITreporting guidelines, and cite them as:

Chan A-W, Tetzlaff JM, Gøtzsche PC, Altman DG, Mann H, Berlin J, Dickersin K, Hróbjartsson A, Schulz KF, Parulekar WR, Krleža-Jerić K, Laupacis A, Moher D. SPIRIT 2013 Explanation and Elaboration: Guidance for protocols of clinical trials. BMJ. 2013;346:e7586

		Reporting Item	Page Number
**Administrative information**			
Title	#1	Descriptive title identifying the study design, population, interventions, and, if applicable, trial acronym	1

For peer review only

Trial registration	#2a	Trial identifier and registry name. If not yet registered, name of intended registry	3
Trial registration: data set	#2b	All items from the World Health Organization Trial Registration Data Set	
Protocol version	#3	Date and version identifier	4
Funding	#4	Sources and types of financial, material, and other support	2, 24
Roles and responsibilities: contributorship	#5a	Names, affiliations, and roles of protocol contributors	23
Roles and responsibilities: sponsor contact information	#5b	Name and contact information for the trial sponsor	24
Roles and responsibilities: sponsor and funder	#5c	Role of study sponsor and funders, if any, in study design; collection, management, analysis, and interpretation of data; writing of the report; and the decision to submit the report for publication, including whether they will have ultimate authority over any of these activities	9
Roles and responsibilities: committees	#5d	Composition, roles, and responsibilities of the coordinating centre, steering committee, endpoint adjudication committee, data management team, and	9 and 17-19

For peer review only

		other individuals or groups overseeing the trial, if applicable (see Item 21a for data monitoring committee)	
**Introduction**			
Background and rationale	#6a	Description of research question and justification for undertaking the trial, including summary of relevant studies (published and unpublished) examining benefits and harms for each intervention	5-6
Background and rationale: choice of comparators	#6b	Explanation for choice of comparators	15-16
Objectives	#7	Specific objectives or hypotheses	6
Trial design	#8	Description of trial design including type of trial (eg, parallel group, crossover, factorial, single group), allocation ratio, and framework (eg, superiority, equivalence, non-inferiority, exploratory)	6-7
**Methods: Participants, interventions, and outcomes**			
Study setting	#9	Description of study settings (eg, community clinic, academic hospital) and list of countries where data will be collected. Reference to where list of study sites can be obtained	9

For peer review only

Eligibility criteria	#10	Inclusion and exclusion criteria for participants. If applicable, eligibility criteria for study centres and individuals who will perform the interventions (eg, surgeons, psychotherapists)	7-8
Interventions: description	#11a	Interventions for each group with sufficient detail to allow replication, including how and when they will be administered	15-17
Interventions: modifications	#11b	Criteria for discontinuing or modifying allocated interventions for a given trial participant (eg, drug dose change in response to harms, participant request, or improving / worsening disease)	14, 21-22
Interventions: adherance	#11c	Strategies to improve adherence to intervention protocols, and any procedures for monitoring adherence (eg, drug tablet return; laboratory tests)	14-17
Interventions: concomitant care	#11d	Relevant concomitant care and interventions that are permitted or prohibited during the trial	15-17
Outcomes	#12	Primary, secondary, and other outcomes, including the specific measurement variable (eg, systolic blood pressure), analysis metric (eg, change from baseline, final value, time to event), method of aggregation (eg, median, proportion), and time point for each outcome. Explanation of the clinical relevance of chosen efficacy and harm outcomes is strongly recommended	10-13

For peer review only

Participant timeline	#13	Time schedule of enrolment, interventions (including any run-ins and washouts), assessments, and visits for participants. A schematic diagram is highly recommended (see Figure)	Table 1, Figure 1
Sample size	#14	Estimated number of participants needed to achieve study objectives and how it was determined, including clinical and statistical assumptions supporting any sample size calculations	9-10
Recruitment	#15	Strategies for achieving adequate participant enrolment to reach target sample size	9, 13-15
**Methods: Assignment of interventions (for controlled trials)**			
Allocation: sequence generation	#16a	Method of generating the allocation sequence (eg, computer-generated random numbers), and list of any factors for stratification. To reduce predictability of a random sequence, details of any planned restriction (eg, blocking) should be provided in a separate document that is unavailable to those who enrol participants or assign interventions	17
Allocation concealment mechanism	#16b	Mechanism of implementing the allocation sequence (eg, central telephone; sequentially numbered, opaque,	17, 18

For peer review only

		sealed envelopes), describing any steps to conceal the sequence until interventions are assigned	
Allocation: implementation	#16c	Who will generate the allocation sequence, who will enrol participants, and who will assign participants to interventions	17
Blinding (masking)	#17a	Who will be blinded after assignment to interventions (eg, trial participants, care providers, outcome assessors, data analysts), and how	7, 16-18
Blinding (masking): emergency unblinding	#17b	If blinded, circumstances under which unblinding is permissible, and procedure for revealing a participant's allocated intervention during the trial	22
**Methods: Data collection, management, and analysis**			
Data collection plan	#18a	Plans for assessment and collection of outcome, baseline, and other trial data, including any related processes to promote data quality (eg, duplicate measurements, training of assessors) and a description of study instruments (eg, questionnaires, laboratory tests) along with their reliability and validity, if known. Reference to where data collection forms can be found, if not in the protocol	10-15, 17

For peer review only

Data collection plan: retention	#18b	Plans to promote participant retention and complete follow-up, including list of any outcome data to be collected for participants who discontinue or deviate from intervention protocols	9, 13-15
Data management	#19	Plans for data entry, coding, security, and storage, including any related processes to promote data quality (eg, double data entry; range checks for data values). Reference to where details of data management procedures can be found, if not in the protocol	19
Statistics: outcomes	#20a	Statistical methods for analysing primary and secondary outcomes. Reference to where other details of the statistical analysis plan can be found, if not in the protocol	19-21
Statistics: additional analyses	#20b	Methods for any additional analyses (eg, subgroup and adjusted analyses)	19-21
Statistics: analysis population and missing data	#20c	Definition of analysis population relating to protocol non-adherence (eg, as randomised analysis), and any statistical methods to handle missing data (eg, multiple imputation)	19-21

Methods: Monitoring

Data monitoring: formal committee	#21a	Composition of data monitoring committee (DMC); summary of its role and reporting structure; statement of whether it is independent from the sponsor and	24

For peer review only

		competing interests; and reference to where further details about its charter can be found, if not in the protocol. Alternatively, an explanation of why a DMC is not needed	
Data monitoring: interim analysis	#21b	Description of any interim analyses and stopping guidelines, including who will have access to these interim results and make the final decision to terminate the trial	24
Harms	#22	Plans for collecting, assessing, reporting, and managing solicited and spontaneously reported adverse events and other unintended effects of trial interventions or trial conduct	12, 14-15, 21-22
Auditing	#23	Frequency and procedures for auditing trial conduct, if any, and whether the process will be independent from investigators and the sponsor	24
**Ethics and dissemination**			
Research ethics approval	#24	Plans for seeking research ethics committee / institutional review board (REC / IRB) approval	23
Protocol amendments	#25	Plans for communicating important protocol modifications (eg, changes to eligibility criteria, outcomes, analyses) to relevant parties (eg, investigators, REC / IRBs, trial participants, trial registries, journals, regulators)	23

For peer review only

Consent or assent	#26a	Who will obtain informed consent or assent from potential trial participants or authorised surrogates, and how (see Item 32)	14
Consent or assent: ancillary studies	#26b	Additional consent provisions for collection and use of participant data and biological specimens in ancillary studies, if applicable	14
Confidentiality	#27	How personal information about potential and enrolled participants will be collected, shared, and maintained in order to protect confidentiality before, during, and after the trial	23
Declaration of interests	#28	Financial and other competing interests for principal investigators for the overall trial and each study site	24
Data access	#29	Statement of who will have access to the final trial dataset, and disclosure of contractual agreements that limit such access for investigators	23
Ancillary and post trial care	#30	Provisions, if any, for ancillary and post-trial care, and for compensation to those who suffer harm from trial participation	24
Dissemination policy: trial results	#31a	Plans for investigators and sponsor to communicate trial results to participants, healthcare professionals, the public, and other relevant groups (eg, via publication, reporting in results databases, or other data sharing arrangements), including any publication restrictions	24

For peer review only

Dissemination policy: authorship	#31b	Authorship eligibility guidelines and any intended use of professional writers	24
Dissemination policy: reproducible research	#31c	Plans, if any, for granting public access to the full protocol, participant-level dataset, and statistical code	24

Appendices

Informed consent materials	#32	Model consent form and other related documentation given to participants and authorised surrogates	-
Biological specimens	#33	Plans for collection, laboratory evaluation, and storage of biological specimens for genetic or molecular analysis in the current trial and for future use in ancillary studies, if applicable	12-13

None The SPIRIT checklist is distributed under the terms of the Creative Commons Attribution License CC-BY-ND 3.0. This checklist can be completed online using https://www.goodreports.org/, a tool made by the EQUATOR Network in collaboration with Penelope.ai

For peer review only

BMJ Open

Efficacy of Metamizole versus Ibuprofen and a Short Educational Intervention versus Standard Care in Acute and Subacute Low Back Pain: A Study Protocol of a Randomized, Multicenter, Factorial Trial (EMISI Trial)

Journal:	*BMJ Open*
Manuscript ID	bmjopen-2020-048531.R2
Article Type:	Protocol
Date Submitted by the Author:	31-Aug-2021
Complete List of Authors:	Wertli, Maria M; University of Bern, Department of General Internal Medicine<br>Flury, Julian S.; University of Bern, Department of General Internal Medicine<br>Streit, Sven; University of Bern, Institute of Primary Health Care (BIHAM)<br>Limacher, Andreas; Universitat Bern Institut fur Sozial- und Praventivmedizin<br>Schuler, Vanessa; University of Bern, Department of General Internal Medicine<br>Ferrante, Asha-Naima; University of Bern, Department of General Internal Medicine<br>Rimensberger, Caroline; University of Bern, Department of General Internal Medicine<br>Haschke, Manuel; University of Bern, Clinical Pharmacology and Toxicology
**Primary Subject Heading**:	General practice / Family practice
Secondary Subject Heading:	Evidence based practice, Emergency medicine
Keywords:	PAIN MANAGEMENT, Back pain < ORTHOPAEDIC & TRAUMA SURGERY, NEUROPHYSIOLOGY

SCHOLARONE™
Manuscripts

I, the Submitting Author has the right to grant and does grant on behalf of all authors of the Work (as defined in the below author licence), an exclusive licence and/or a non-exclusive licence for contributions from authors who are: i) UK Crown employees; ii) where BMJ has agreed a CC-BY licence shall apply, and/or iii) in accordance with the terms applicable for US Federal Government officers or employees acting as part of their official duties; on a worldwide, perpetual, irrevocable, royalty-free basis to BMJ Publishing Group Ltd ("BMJ") its licensees and where the relevant Journal is co-owned by BMJ to the co-owners of the Journal, to publish the Work in this journal and any other BMJ products and to exploit all rights, as set out in our licence.

The Submitting Author accepts and understands that any supply made under these terms is made by BMJ to the Submitting Author unless you are acting as an employee on behalf of your employer or a postgraduate student of an affiliated institution which is paying any applicable article publishing charge ("APC") for Open Access articles. Where the Submitting Author wishes to make the Work available on an Open Access basis (and intends to pay the relevant APC), the terms of reuse of such Open Access shall be governed by a Creative Commons licence – details of these licences and which Creative Commons licence will apply to this Work are set out in our licence referred to above.

Other than as permitted in any relevant BMJ Author's Self Archiving Policies, I confirm this Work has not been accepted for publication elsewhere, is not being considered for publication elsewhere and does not duplicate material already published. I confirm all authors consent to publication of this Work and authorise the granting of this licence.

For peer review only

Efficacy of Metamizole versus Ibuprofen and a Short Educational Intervention versus Standard Care in Acute and Subacute Low Back Pain: A Study Protocol of a Randomized, Multicenter, Factorial Trial (EMISI Trial)

Maria M. Wertli[1], Julian S. Flury[1], Sven Streit[2], Andreas Limacher[3], Vanessa Schuler[1], Asha-Naima Ferrante[1,4], Caroline Rimensberger[1], Manuel Haschke[5]

[1]Department of General Internal Medicine, Inselspital, Bern University Hospital, University of Bern, Bern, Switzerland

[2]Institute of Primary Health Care (BIHAM), University of Bern, Bern, Switzerland.

[3]CTU Bern, University of Bern, Bern, Switzerland

[4]Faculty of Human Sciences and Department of Psychology, University of Bern, Bern Switzerland

[5]Clinical Pharmacology and Toxicology, Department of General Internal Medicine, Inselspital, Bern University Hospital, University of Bern, Switzerland

Corresponding author

Maria Wertli, MD PhD

Department of General Internal Medicine

Bern University Hospital

Freiburgstrasse 8

3010 Bern

Mail: Maria.Wertli@insel.ch

Phone: +41 31 632 01 46

For peer review only

Abstract

INTRODUCTION Low back pain (LBP) is among the top three most common diseases worldwide, resulting in a life with pain-related disability. To date, no study has assessed the efficacy of metamizole (dipyrone), a non-opioid analgesic and antipyretic prodrug compared to the conventional NSAID ibuprofen, in patients with an acute low back pain episode. Further, it is unclear, whether a short-educational intervention is superior to usual care alone.

OBJECTIVES The objective of this study is to assess first, whether metamizole is non-inferior to ibuprofen in a new episode of acute or subacute LBP. Second, we aim to assess whether a short educational intervention including evidence-based patient information on the nature of LBP is superior to usual care alone.

METHODS AND ANALYSIS An investigator-initiated multicenter, randomized, double blind trial using a factorial design will be performed. A total of 120 participants with a new episode of LBP will be recruited from GP practices, outpatient clinics, and from emergency departments, and randomized into four different treatment groups: ibuprofen alone, ibuprofen and short-intervention, metamizole alone, metamizole and short-intervention. The primary endpoint for the medical treatment will be change in pain assessed on an 11-point numeric rating scale (NRS) after 14 days. The primary outcome for the short intervention will be change in the Core Outcome Index (COMI) assessed after 42 days.

ETHICS, DISSEMINATION, AND FUNDING This study has been approved by the responsible Ethics Board (Ethikkommission Bern / 2018-01986) and the Swiss Agency for Therapeutic Products (Swissmedic / 2019DR4002). Results will be published in open access policy peer-reviewed journals. The study is funded by the Swiss National Science Foundation (grant number 32003B_179346).

For peer review only - http://bmjopen.bmj.com/site/about/guidelines.xhtml

TRIAL REGISTRATION NCT04111315

For peer review only

For peer review only - http://bmjopen.bmj.com/site/about/guidelines.xhtml

Article Summary

Strengths and limitations of this study

- This is the first randomized double-blind study assessing the analgesic effect of metamizole compared to the conventional NSAID ibuprofen in patients with acute or subacute LBP presenting to the GP or emergency department.
- In addition, this study will assess whether a short educational intervention is more effective in LBP patients compared to usual care.
- Due to the high proportion of patients who will not require extended pain treatment during an acute LBP episode, the trial design mirrors current clinical practice.
- The primary endpoint pain intensity will be assessed after 14 days. The primary outcome for the short intervention will be assessed after 42 days.
- While patients, physicians, and researchers are blinded to the pharmacological treatment, blinding is not feasible for the short educational intervention.

Keywords

low back pain, randomized controlled trial, pain medication ibuprofen, NSAID, metamizole, psychological intervention, outcome

Word count:

5631 words.

Protocol Version 2, July 1. 2021

For peer review only

Introduction

Low back pain (LBP) is among the top three most common diseases worldwide, resulting in a life with pain-related disability.[1] In patients with persistent LBP over 3 months, the risk for chronic pain increases sharply[2] and effective interventions should aim to prevent pain persistence without overtreatment[3]. Therefore, treatment guidelines recommend the use of pain medication and to keep patients physically active during an acute LBP episode.[4 5] Despite the frequency of LBP, only few high-quality studies assessed the efficacy of pain medication. Systematic reviews of randomized clinical trials found opioids to be no more effective than non-opioid medication in LBP[6 7] but opioids were associated with potentially severe side-effects.[4 7 8] Therefore, non-opioid pain medications that are safe and effective are urgently needed to control pain.

Current treatment guidelines recommend non-steroidal anti-inflammatory drugs (NSAIDs) for acute pain control.[4 9] However, adverse events including kidney injury and gastrointestinal bleeding limit their use in many patients. Metamizole (dipyrone) is a non-opioid analgesic and antipyretic with a favorable gastrointestinal and renal safety profile.[10] For example, metamizole use was associated with a lower risk for gastrointestinal bleeding compared to NSAIDs and for hepatotoxicity compared to paracetamol.[11] Although Metamizole has been withdrawn from the market or never approved in many countries including the USA, Canada, and UK,[12] it is frequently used in many other countries such as Germany,[13] Spain,[12] Switzerland,[14] some Eastern European, and South American countries.[12 15] Despite its increasing use in many European countries,[14 16] the role of metamizole for the treatment of LBP is unclear and has so far not been systematically studied. This may be due to an ongoing controversy regarding the risk of metamizole-associated agranulocytosis.[12 15] Due to the rare occurrence, incidence rates have been based on case control studies and pharmacovigilance data. The incidence of the metamizole induced agranulocytosis was

For peer review only - http://bmjopen.bmj.com/site/about/guidelines.xhtml

estimated between 0.5 and 3.5 cases per 1 million metamizole users over one week per year.[17-28] Even though the overall risk of agranulocytosis is higher compared to other analgesics, it remains a rare event occurring only in a small proportion of susceptible patients[17-25 27-29] and the overall safety profile of metamizole is still favorable compared to NSAIDs or opioids.[30]

Many patients have reservations against regular intake of pain medication and, contrary to guideline recommendations, limit physical activity to keep pain manageable without medication.[31] Patient education encouraging physical activity may help to improve pain control and function. For example, a Cochrane review found that 2.5-hour educational sessions were more effective than no intervention on the return-to-work rate in patients with acute/subacute LBP.[32] Such time-intensive interventions may not be necessary in all patients and are not feasible in a busy primary care practice. Short educational interventions would be more practical, however, to date it is unclear whether they also have a positive effect on outcome in patients with a new LBP episode.

Objectives

The first objective of this study is to assess whether metamizole is non-inferior to ibuprofen in patients with a new episode of acute or subacute LBP. The second objective is to assess whether a short educational intervention, including evidence-based patient information on the nature of LBP, is superior to usual care alone. Thus, this randomized trial will provide new knowledge regarding the efficacy of non-opioid pain medications in LBP. In addition, if the short educational intervention proves to be beneficial compared to usual care, the trial will help to improve care for patients with a new LBP episode.

For peer review only - http://bmjopen.bmj.com/site/about/guidelines.xhtml

Methods and analysis

The methods reporting of this randomized trial will follow the recommendations of the SPIRIT statement.[33]

Design: Multicenter, randomized, double-blind trial using a factorial design

The EMISI study is a multicenter, randomized, double-blind trial using a factorial design to investigate whether metamizole is non-inferior to ibuprofen in patients with a new onset LBP episode. Patients, physicians, and researchers will be blinded to the allocation of the study medication. A non-inferiority design was chosen as metamizole seems to be equally effective as ibuprofen in clinical practice. Furthermore, the study will assess whether a short educational intervention including evidence-based patient information is superior to usual care. Blinding patients about the short intervention is not possible but blinding is upheld in GPs and researchers (double blind). The primary evaluation of outcomes (see Outcome measure for details) will be performed at day 14 (comparison of metamizole to ibuprofen) and at day 42 (comparison of educational short intervention to usual care).

Eligibility criteria

Subjects fulfilling all of the following inclusion criteria are eligible for the study:

- Age 18 years or older
- Seeking care for new onset of non-specific or specific LBP (pain duration of less than 12 weeks LBP prior to the baseline visit)
- The GP plans to prescribe a non-opioid pain medication for pain control
- Informed consent documented by signature

For peer review only - http://bmjopen.bmj.com/site/about/guidelines.xhtml

Exclusion criteria are:

- Chronic use of opioids
- Presence of red flags (serious neurological deficit requiring surgery, infection, vertebral fracture)
- Known intolerance to the study medication (i.e. previous acute allergic reaction to NSAIDs or metamizole)
- Active malignancy and / or history of a (previous) hematologic disorder (history of agranulocytosis)
- History of anemia (hemoglobin of <100.0 mg/L), neutropenia (leucocyte count of < 3.0G/L), thrombocytopenia (<100G/L)
- Known contraindications against the study medications: previous gastrointestinal ulcer/bleeding, inflammatory bowel disease, heart failure (NYHA III-IV), liver failure (liver cirrhosis, ascites), renal insufficiency (estimated glomerular filtration rate (eGFR) < 60 ml/min/1,73 m2) or previous acute kidney injury (AKI stage 2 according to the KDIGO definition)
- Immune deficiency or under immunosuppressant treatment
- Known or suspected non-compliance, drug or alcohol abuse
- Participation in another study within 30 days preceding randomization and during the present study or previous enrolment into the current study
- Enrolment of the investigator, his/her family members, employees, and other dependent persons
- Pregnancy: In women of childbearing age, a negative pregnancy test (urine or blood test as available in the primary care practice) before inclusion is required. Women who are not willing to use safe contraception (condom or birth control pill)

For peer review only - http://bmjopen.bmj.com/site/about/guidelines.xhtml

during the trial, intention to become pregnant during the trial, pregnancy, or breast feeding.

Study setting

The EMISI trial will be coordinated by the central study team at the Division of General Internal Medicine, University Hospital Bern, Switzerland. Patients will be recruited from outpatient clinics, from GP practices, and from emergency departments. The overall study duration of 42 days (6 weeks) was chosen because treatment guidelines recommend further assessment of patients for multidisciplinary treatment if LBP persists for more than 6 weeks despite pain medication and the advice to stay active. This is a investigator initiated trial. The funder will have no role in the study design, the trial oversight, data collection, analysis of the study, and publication of the results.

Estimated sample size and power

Both interventions are considered for determining the sample size for this factorial trial.[34] We expect the two primary hypotheses to be independent of each other and do not expect an interaction between both interventions. Therefore, we powered each hypothesis individually, but accounted for multiple testing by adjusting the type-I-error rate by setting the alpha level to 0.025 in each test to keep the overall type-I error rate at 0.05. Thus, we aimed to include 120 patients to account for a drop-out of 10% which will allow to proof non-inferiority of the metamizole treatment at a one-sided alpha-level of 2.5% with a power of 90%.

For peer review only - http://bmjopen.bmj.com/site/about/guidelines.xhtml

The sample size calculation for the comparison of metamizole vs. ibuprofen was based on a difference in the change of pain score in the numeric rating scale (NRS; range 0 to 10, higher score indicates more pain). We considered a change of ≥2 points between the two groups as clinically relevant and a change of ≤1 point as negligible.[35 36] Therefore, we set the non-inferiority margin to -1 score point. Based on a two-sample means test and a standard deviation of 1.6,[35] we will need 108 patients (54 per group) to proof non-inferiority of the metamizole treatment at a one-sided alpha-level of 2.5% with a power of 90%.

The sample size for the primary outcome comparison short intervention vs. usual care (superiority) is based on a change in Core Outcome Measures Index (COMI; range 0 to 10, higher score indicates higher level of complaint, see primary outcomes) from baseline to week 6. We hypothesize that there is a difference between the two intervention groups regarding COMI. Mannion et al.[37] reported a minimal clinically important difference for improvement in the COMI of 2.2 points and standard deviations for changes in COMI ranging from 1.7 to 2.5 points. We see a difference of 2.2 points between the two groups as clinically relevant and assume a standard deviation of 2.2 points. Based on a two-sample means test, we will need 54 patients (27 per group) to detect a difference in the change of the COMI at a two-sided alpha-level of 2.5% with a power of 90%.

Primary outcome

Table 1 provides an overview of measurements according to the Standard Protocol Items: Recommendations for Interventional Trials. The primary outcome for the comparison of metamizole vs. ibuprofen is the change in pain on the NRS (range 0-10 points) from baseline to follow-up at day 14 (non-inferiority). Pain intensity will be recorded as average pain intensity for the past 24 hours on an NRS scale from 0 to 10,

For peer review only - http://bmjopen.bmj.com/site/about/guidelines.xhtml

scored from 0 (no pain) to 10 (worst possible pain). The rational for assessing the effectiveness outcome at 14 days was based on the double-blind RCT comparing paracetamol to placebo.[38]

The primary outcome for the comparison of the short intervention vs. usual care is the change in the COMI sum-score (range 0-10) from baseline to 42 days follow-up (superiority). The COMI is a short, multidimensional, validated outcome instrument with excellent psychometric properties recommended for the monitoring of the outcome in patients undergoing treatment for LBP.[37] The COMI sum score is calculated as described in the validation paper.[39 40] The pain scales (item 2a and 2b) are scored 0-10, whilst the category scales (items 3-7) are scored as 0, 2.5, 5.0, 7.5, 10.0. The sum score (0-10) for the whole COMI scale is calculated based on the following 5 domains:

- Pain domain: higher of the two pain scores (higher of 2a and 2b) is taken as the score
- Disability domain: the average of the two disability items (6 and 7)
- Back related function (item 3)
- Symptom specific well-being (item 4)
- General quality of life (item 5)

The five domain scores are then averaged to give a COMI score from 0-10.

Two additional questions cover the satisfaction with treatment outcome. A minimal clinically important difference (MCID) found to be relevant for patients was a reduction in COMI score of ≥2.2 points.[37]

Secondary outcome

All outcome measures used in this study were recommended by the NIH Task force and other expert panels to study back pain.[41 42] **Table 1** provides an overview of

For peer review only - http://bmjopen.bmj.com/site/about/guidelines.xhtml

measurements according to the Standard Protocol Items: Recommendations for Interventional Trials.[33]

Secondary outcome measures include:

- Change in pain on the numeric rating scale (NRS, range 0-10 points) from baseline to follow-up at day 42
- Change in the Core Outcome Measures Index (COMI) sum-score (range 0-10) from baseline to 14 days follow-up
- The proportion of patients with a minimal clinically important difference (MCID) in the NRS (≥2 points) and COMI (≥2.2 points) at days 14 and 42
- Time to recovery (recovery defined as the first day with a NRS of <2 points sustained for 2 consecutive days)
- Change in overall pain intensity from baseline to follow-up at day 14 and day 42. Overall pain intensity: average of ratings for average pain, pain at rest, and pain during activity during the last 24 hours.
- Area under effect curve (AUEC day 0 to day 14) NRS
- Adverse events (AE)
- Time to stopping treatment
- Global effectiveness of the treatment (COMI question ‘‘how much did the operation/treatment help your back problem?’’ 5-point Likert scale from ‘‘helped a lot’’ to ‘‘made things worse’’)
- Daily physical activity (daily step count)
- Use of pain medication and additional rescue medication
- Additional treatment (physical therapy, injections)
- Return to work (yes / no)

For peer review only - http://bmjopen.bmj.com/site/about/guidelines.xhtml

- Treatment satisfaction (COMI)
- Quality of life as assessed by the 5-level version of the European Quality of Life-5 Dimensions questionnaire (EQ-5D)
- Psychological factors (STarT-Back Tool)[43]

Exploratory variables

Response to pain medications may be influenced by individual pharmacokinetic and pharmacodynamics factors. Therefore, we will collect blood samples during visit 2 (day 14) to assess the following factors that may influence the individual response to the pain medications. First, we will measure plasma concentrations of ibuprofen and the four main metamizole metabolites (4-MAA, 4-AA, 4-FA, 4-AAA). Pharmacodynamic effects of metamizole correlate primarily with concentrations of the 4-MAA metabolite,[44] thus direct quantification of 4-MAA concentrations will add important information for the interpretation of the observed effects.[45]

Second, as individual response to ibuprofen may be affected by genetic polymorphisms of CYP isoforms 2C8 and 2C9, the study population will be characterized in terms of cytochrome P450 (CYP) 2C8/9 genotypes (CYP2C8 *1 and *3 alleles, CYP2C9 *1, *2, and *3 alleles). In subjects with alleles conferring low CYP2C8 and / or CYP2C9 activity, reduced ibuprofen clearance of both enantiomers has been described, resulting in several-fold increased exposure and prolonged half-life compared to subjects with wild-type alleles.[46 47]

Study procedures

Study procedures are summarized in the study flow chart (**Figure 1**). Each participating site will receive a supply of sequentially coded, blinded, and sealed

For peer review only - http://bmjopen.bmj.com/site/about/guidelines.xhtml

intervention packs. All patients presenting with LBP will be screened for potential inclusion into the study. After obtaining informed consent by the physician, patient information will be entered in a web-based clinical data management system (CDMS). After patient registration in the CDMS and confirmation of all inclusion and exclusion criteria, patients are randomized using sequentially coded and blinded intervention packs, thus ensuring concealment of allocation. The number of the intervention pack will be recorded in the CDMS.

Patients will complete a set of self-reported questionnaires (see Table 1 for details) on demographics (age, sex, work, and civic status), previous history, current medications, pain related information (NRS, pain drawing), disability (COMI, STarT Back tool), quality of life (European Quality of Life-5 Dimensions questionnaire (EQ5-D-5L), the Fear Avoidance Questionnaire (FABQ),[48] self-efficacy (Schmerzspezifische Selbstwirksamkeit (FESS[49])). Physicians will complete a questionnaire on clinical findings, the clinical diagnosis, previous and newly prescribed treatments.

Upon completion of the baseline evaluation, patients may open the intervention pack and start treatment. The intervention pack will contain a pill bottle and an envelope with the assignment of the short vs no intervention, and a pain diary.

Between the first and the second visit, patients will record their pain before intake of study drug, the number of study drugs and additional pain medications, and their daily physical activity (quantified by daily step counts using a commercially available wearable step tracking device (Garmin vivofit 4®).

To mitigate the risk for AEs, we will collect blood samples after the randomization in all patients. In case of a newly detected renal insufficiency (defined as estimated glomerular filtration rate (eGFR) < 60 ml/min/1.73 m^2), leucopenia (leucocyte count <

For peer review only - http://bmjopen.bmj.com/site/about/guidelines.xhtml

3.0G/L), thrombocytopenia (defined as <100 G/L) and/or anemia ((hemoglobin <100g/L), we will stop the study medication.

During the second visit (day 14), patients will return the pill bottles and the pain diary to the GPs office where they are collected for pill count. The patient will receive a new drug pack and pain diary. A blood sample will be collected (creatinine, blood count, drug concentrations and genotyping), and self-reported questionnaires and a clinical assessment will be completed. The GP will record the clinical findings and additional pharmacological and non-pharmacological treatments.

To improve adherence, study staff will call patients between the first and the second visit and between the second and the final visit and ask for adverse events and additional pain treatment. As we expect a high proportion of patients to have recovered by day 28 (second phone call), researchers will then determine whether a clinical visit on day 42 is necessary. In case patients have recovered by day 28, patients do not need to attend the clinical visit. Patients will be asked whether they would be willing to participate in an in-person interview at the end of the study. The aim of the interview is to assess factors that were relevant for patients during their LBP treatment. The interview will be recorded (video and audio) for qualitative analysis using standard grounded theory method according to Philipp Mayring.[50]

A final assessment will be done on day 42 during a clinical visit in patients who had persisting symptoms on day 28. Patients will return the pain diary and the pill bottles with the remaining pills to the GPs office where they are collected for pill count. Data collection includes self-reported validated questionnaires. The GP will record the clinical findings and additional pharmacological and non-pharmacological treatments. A final blood sample (serum creatinine and blood count) will only be collected if patients are still taking study medication. Patients who do not need to attend the

For peer review only - http://bmjopen.bmj.com/site/about/guidelines.xhtml

clinical visit will receive a final identical set of questionnaires by mail and will be asked for AE/SAE and additional treatments during a phone call. Patients will return the questionnaires together with the pain diary and the pill bottles to the central study team. To assess the success of blinding, patients will be asked at the end of the study what treatment group they believe they had been assigned to (metamizole or ibuprofen).

Pharmacological intervention

During the first four days, we will compare a fixed-dose regimen of metamizole 500mg to 500 mg ibuprofen lysine (lysine-2-[4-isobutylphenyl]-propionate, corresponds to 292.6 mg ibuprofen acid) three times two capsules per day (**Table 2**). Ibuprofen was chosen as a comparator because it is a well-studied and the most frequently prescribed NSAID in Switzerland[14] with a similar dosing interval as metamizole. The initial dose of both medications is the currently used and recommended dose for the treatment of acute pain (common daily dose for metamizole 3000mg (maximum daily dose 4000mg) and 3000mg ibuprofen lysine (maximum daily dose 4000mg)). Ibuprofen lysine 3000mg corresponds to 1800mg ibuprofen acid. The ibuprofen lysine formulation was chosen to facilitate blinding of the study drug, as ibuprofen lysine 500mg was the only ibuprofen formulation available on the Swiss market, which could be prescribed with identical number of capsules and at identical intervals as metamizole. The lysine salt of ibuprofen shows better solubility and faster absorption compared with the acid form.[44 51] However, overall extent of absorption and analgesic effect in an acute pain model did not differ from the acid form.[52]

In LBP, current clinical practice is to prescribe an initial fixed dose regimen followed by an as-needed regimen that allows patients to adjust the daily dose depending on their pain intensity. To mirror clinical practice, patients will be allowed after the first four days

For peer review only - http://bmjopen.bmj.com/site/about/guidelines.xhtml

to decrease the intake of the study medication according to a dose reduction schedule (as needed phase). From day 4 until recovery patients may reduce the daily pain medication intake once their pain has decreased to less than 4 points on the NRS (range 0-10 points) every two days. The treatment duration will thus depend on the duration of pain. Patients are considered to have recovered when their pain is below 2 (NRS 0-10) on 2 consecutive days after they have stopped intake of pain medication (end of treatment).

Non-pharmacological educational short intervention

Patients assigned to the short intervention will receive together with the pain medication a short leaflet focusing on the nature of non-specific LBP and the importance of staying active. A second leaflet contains exercises to alleviate LBP. The content of the short information leaflet is based on the *The Back Book*.[53] Further, all patients assigned to the short intervention will receive a 10-minute standardized telephone intervention delivered by a trained member of the study team during the first 4 treatment days to help patients understand the non-dangerous nature of LBP, and to explain the purpose of pain medication to facilitate movement. In addition, patients will be encouraged to stay active and to perform the exercises as proposed in the exercise leaflet.

Concomitant interventions

In a double-blind RCT comparing paracetamol to placebo for LBP in the primary care setting, rescue medications were infrequently used.[38] Therefore, physicians will be allowed to prescribe additional rescue medications recommended by treatment guidelines at their discretion. Additional medication use is recorded in the pain diary, during the phone calls, and the clinical visits. Further, information on any use of physical therapy, massage or other treatments is collected during the follow-up visits at days 14

For peer review only - http://bmjopen.bmj.com/site/about/guidelines.xhtml

and 42. Although GPs will be instructed to withhold physical therapy until day 14 (i.e. the primary effectiveness endpoint for metamizole vs. ibuprofen) if the clinical situation allows, additional care including physical therapy and relaxation strategies recommended by guidelines will be at the discretion of the treating GP. GPs will record any additional prescription of addiational care. Concomitant treatments will be treated as co-variates to assess their potential impact on overall efficacy (performance bias).

Randomization and allocation procedures

The allocation sequence (randomization list) will be generated by an independent data manager at the CTU Bern who is not otherwise involved in the trial. Randomization will be blocked and stratified according to study sites. To ensure concealment of allocation, block size will not be communicated. After patient data is registered in the CDMS, eligibility is confirmed and signed informed consent is obtained, patients will be assigned to the next sequentially coded intervention pack that is available at the recruiting site. Patients are randomized 1:1:1:1 into one of four groups: Metamizole + educational intervention vs. metamizole + standard care vs. ibuprofen + educational intervention vs. ibuprofen + standard care. The intervention packs will contain a pill bottle and an envelope with the assignment of the short vs. no intervention. The intervention packs will be prepared according to the randomization list by an independent hospital pharmacist not involved in the study.

Blinding

Staff at the hospital pharmacy not involved in patient recruitment or other trial activities will fill the gelatin capsules with the study medication and label the pill bottles according to a standard operating procedure and based on the randomization list provided by the CTU Bern. The study medication will be delivered in identically labeled sealed pill bottles

For peer review only - http://bmjopen.bmj.com/site/about/guidelines.xhtml

containing capsules of identical appearance. The randomization list will be kept electronically at the CTU Bern in a password-protected file. Study personnel and physicians involved in patient recruitment, treatment, and outcome assessment will have no access to the list.

Due to the nature of the short educational intervention, no blinding of patients receiving an information leaflet and a telephone call is possible. Therefore, contamination and performance bias are important to consider. The envelope including the leaflet will be sealed and the patients will be instructed to open de envelope outside the GPs office. GPs will not be informed about the group assignment and the content and aim of the educational intervention. Patients assigned to the educational intervention will receive a telephone call according to a predefined script by a member of the study team otherwise not involved in patient recruitment, outcome assessment, and data analysis. To minimize the risk of detection bias, the study outcomes are collected in pain diaries and self-reported questionnaires (NRS at day 14, COMI at day 42). Study personnel that will contact patients by phone for reminders and to collect information on adverse events will be blinded to the group assignment. To assess differences in additional treatments (performance bias), patients and GPs will record additional treatments other than the study medication and the educational information (e.g. additional medication use, physical therapy, injections).

The statistician responsible for the final analysis will also have no access to the list until the primary analysis of the trial is finished. Data analysis will be done according to a prespecified analysis plan before the randomization code will be broken. Patients, physicians, and data analysts will be blinded with regard to the allocation to the study medication until after the primary analysis. To assess the success of blinding, we will ask patients at the end of the study into which group they belief they had been assigned to. Data on all recruited patients will be collected and regularly monitored.

For peer review only - http://bmjopen.bmj.com/site/about/guidelines.xhtml

Statistical analysis plan

The statistical analysis will be performed in compliance with the International Conference on Harmonisation's Good Clinical Practice guidelines and the report will be developed in line with the Consolidated Standards of Reporting Trials statement.

To assess the efficacy for the two primary outcomes, we will use two analysis sets (see **Figure 1**). The full analysis set (FAS) includes all randomized patients for the intention-to-treat (ITT) analysis, i.e. all patients will be analyzed in the group they were assigned to. The *per protocol (PP) set* is based on the FAS, excluding patients with major protocol violations, patients who were excluded due to the predefined stop criteria, patients withdrawing from the trial within 3 days of treatment start, patients undergoing surgery, and patients that did not receive the randomized treatment or intervention.

We will present patient and procedural characteristics using descriptive statistics (mean and standard deviation or median and interquartile range for continuous variables and frequency and percentage for categorical variables).

To compare the efficacy of metamizole and ibuprofen (non-inferiority), we use an ITT (FAS set) and a PP analysis (PP set) for the primary outcome (NRS change from baseline to follow-up at 14 days). Both analyses need to meet non-inferiority in order to claim success. The difference in the change in NRS between the two groups will be calculated from a linear model, adjusted for the baseline NRS value and the type of intervention (short intervention vs standard care). If the lower one-sided 97.5% confidence limit of the difference lies above the non-inferiority margin of -1, non-inferiority will be claimed. If we can demonstrate non-inferiority, we will additionally test for superiority at a two-sided significance level of 2.5%. Since testing is nested (i.e. hierarchical), this procedure has no implication on the overall type-I error rate.

For peer review only - http://bmjopen.bmj.com/site/about/guidelines.xhtml

To compare the primary outcome between the short educational intervention to usual care (superiority), we use ITT analysis (COMI change from baseline to day 42). The difference in the change in COMI between the two groups will be calculated and tested at a two-sided significance level of 2.5% using a linear model, adjusted for the baseline COMI and the type of drug treatment. Differences will be presented with a two-sided 97.5% confidence interval (CI) and the regular 95% CI.

We will assess both primary outcomes for interaction. We will introduce an interaction term between the treatment indicator and the intervention indicator to test whether in A) the effect of the metamizole treatment on the change in NRS at day 14 is different in patients that receive the short intervention compared with the standard care group and in B) whether the effect of the short intervention on the change in COMI at day 42 is different in patients that receive metamizole compared with patients that receive ibuprofen.

Secondary continuous outcomes will be analyzed as the primary outcomes. Besides the indicator for the drug treatment or intervention, models will be adjusted for the baseline value if available. Secondary binary outcomes will be analyzed using logistic regression models adjusted for the indicator for the drug treatment or intervention, as applicable. Time-to-event outcomes will be analyzed using Cox regression adjusted for the indicator for the drug treatment or intervention, as applicable. For all secondary effect measures, we will display regular two-sided 95% CI and accompanying p-values.

Further, we will perform additional PP analyses for the primary outcome of the comparison between the two intervention arms as well as for all secondary outcomes. If outcomes are missing, we will employ multiple imputation in the primary analysis and additionally perform an available case analysis disregarding missing data. Continuous outcomes that are measured several times during follow-up will additionally be evaluated

For peer review only - http://bmjopen.bmj.com/site/about/guidelines.xhtml

in a repeated measures mixed-effects linear model, additionally introducing a random intercept for patients into the model.

Exploratory analysis will be conducted with regards to predictors for treatment responses. We will explore the association between clinical predictors (e.g. depression, fear avoidance beliefs, social factors), drug levels, or genetic mutations and the treatment response in linear models. We will perform subgroup analyses according to gender, genetic phenotypes, and drug levels.

Safety outcomes will be reported as proportion and 95% CI separately for the two drug treatment groups using the safety population (i.e. all patients that have received at least one dose of the study drug and at least one safety-related visit or observation) set.

Patients with missing outcome data will be imputed using multiple imputation.

Safety

Protocol violations should not lead to treatment discontinuation unless they indicate a significant risk to patient safety.

Patients may be withdrawn from the study treatment for the following reasons:

- Hematologic disorders including leucopenia (defined as leucocyte count < 3.0G/L; thrombocytopenia (defined as <100 G/L), and anemia (decrease in hemoglobin ≥20g/L or <100g/L)
- Potential evidence for a hematologic disease (pathological blood smear needing further evaluation)
- Decreased kidney function (defined as an estimated creatinine clearance using the MDRD formula of < 50 ml/min/1.73m^2) or acute kidney injury stage 1 or higher

For peer review only - http://bmjopen.bmj.com/site/about/guidelines.xhtml

(defined according to the KDIGO definition: ≥1.5 times creatinine increase compared to baseline value or ≥26.5 micro mol/L creatinine increase)

- Development of toxicity which, in the investigators or GPs judgment, precludes further use of one of the study drugs
- Patients lost to follow-up or non-compliance

We will comply with all regulations concerning safety measures in clinical trials as set forth by the Swiss Agency for Therapeutic Products (Swissmedic). The investigators will report any serious adverse events occurring during clinical trials, independent of direct causal relationship with the treatment, within 24 hours. In case of a suspected unexpected serious adverse reaction (SUSAR), Swissmedic will be notified by the sponsor-investigator within the legal timelines. Unblinding will be permissible in case the information is required to ensure the patients safety in case of an AE.

All adverse events (AE) are reviewed by a safety committee and graded according to the National Health Service (NIH) Common Terminology Criteria for Adverse Events (CTCAE v5.0).[54]

Patient and public involvement

In this study, we use information material developed by patient advocacy groups. The results of this study will be communicated to the patients and physicians participating in the trial. The results will also be communicated to patients' advocacy groups and may help to improve the information material. Further, we will assess the impact of the study on patients and factors that may be facilitators or barriers to improve pain and pain management in LBP during in person interviews.

For peer review only - http://bmjopen.bmj.com/site/about/guidelines.xhtml

Clinical implications

During an LBP episode treatment guideline recommend to alleviate pain using pain medication and to stay physically active. To alleviate pain, NSAIDs are frequently used. However, the use of NSAIDs may be limited because of common adverse effects. Metamizole has a favorable adverse effects profile and may be used as an alternative. However, there is a lack of data on its effectiveness and safety for the treatment of LBP. This is the first study that systematically investigates the efficacy of metamizole in LBP patients undergoing treatment over the course of several weeks. Therefore, this study will provide evidence to support or discourage the use of metamizole in LBP. The findings of this study will be especially relevant for those patients where NSAIDs should not be used. Moreover, this trial is expected to provide evidence for a highly needed non-opioid treatment alternative in musculoskeletal pain. In particular, because the use of opioids has been shown to be no more effective than non-opioid medication[55] and is associated with potentially severe adverse events.

Non-pharmacological educational measures may potentiate the effect of the pain medication in acute LBP by addressing misbeliefs and avoiding disuse. Despite good evidence that staying active during an acute LBP episode is more effective than bed rest,[56] more than 50% of the Swiss population report avoiding movements and increasing rest during a LBP episode.[57] The interaction between patient and doctor early on influences the recovery of patients and many patients may benefit from a short educational intervention that is easy to be implemented even in a busy GP practice in addition to pain medication. This is the first study that investigates the impact of a short educational intervention in addition to pain medication on the recovery and medication adherence in patients with acute and subacute LBP. Even a small individual effect may have a large impact on the overall societal burden of LBP. Our work will inspire novel approaches on how to improve adherence to medication and behavioral changes.

Ethics and dissemination

The trial will be performed in compliance with the Declaration of Helsinki and its amendments, and has been approved by the responsible Ethics Board (Ethikkommission Bern/ 2018-01986) and the Swiss Agency for Therapeutic Products (Swissmedic / 2019DR4002). Important protocol modifications will be communicated to

For peer review only - http://bmjopen.bmj.com/site/about/guidelines.xhtml

the Ethics Board and participating study sites. Potential participants must provide written informed consent before entering the study. Subjects can leave the study at any time for any reason without any negative consequences for their further treatment. When a patient ends the treatment phase of the study prematurely, we will record the date and reason for early treatment discontinuation. If possible, the end of treatment evaluations will be collected before the patient is started on any other therapeutic intervention. Insurance coverage will be provided for all study participants by the study sponsor.

The results of the main trial and each of the secondary outcomes will be submitted for publication in open access peer-reviewed journals. Results will be disseminated to all participating centers and participants expressing interest. The EMISI study group is an independent academic research group, which will not employ professional writers. The EMISI study group will comply with the open access regulation of the Swiss National Science Foundation.

Authors' contributions

MMW, SS, AL, JF, and MH designed the study. AF, VS and CR critically commented on the methods and contributed to the development of the study. MMW and JF wrote the first draft of the manuscript. All authors revised the manuscript and approved the final version.

Funding statement

Efficacy of Metamizole versus Ibuprofen and a Short Educational Intervention versus Standard Care in Acute and Subacute Low Back Pain: A Randomized, Factorial Trial (The EMISI Trial) is supported by the Swiss National Science Foundation (grant number:

For peer review only - http://bmjopen.bmj.com/site/about/guidelines.xhtml

32003B_179346 / 1). This research project received no further support from any other funding agency.

Competing interests

Drs. Haschke and Wertli received a grant from the Swiss Science National Foundation for the study: grant number 32003B_179346. The funder plays no role in the study design, collection of data, analysis, and writing of the manuscript. There is no conflict of interest regarding intellectual content and proprietary affairs.

Data monitoring committee

Data monitoring will be conducted by the CTU Bern. Data collection, handling of the data, and the analysis will be done by the researchers at the University of Bern. The funder plays no role in the data acquisition, data storage, and analysis.

There is no interim analysis planned. Auditing will be performed by an independent auditor of the CTU at the central study site.

Trial sponsor investigator

Maria Wertli, MD PhD
Department of General Internal Medicine
Bern University Hospital
Freiburgstrasse 8
3010 Bern
Mail: Maria.Wertli@insel.ch
Phone: +41 31 632 01 46

For peer review only - http://bmjopen.bmj.com/site/about/guidelines.xhtml

Bibliography

1. Vos T, Allen C, Arora M, et al. Global, regional, and national incidence, prevalence, and years lived with disability for 310 diseases and injuries, 1990-2015: a systematic analysis for the Global Burden of Disease Study 2015. Lancet (London, England) 2016;**388**(10053):1545-602.
2. da C Menezes Costa L, Maher CG, Hancock MJ, et al. The prognosis of acute and persistent low-back pain: a meta-analysis. CMAJ: Canadian Medical Association journal 2012;**184**(11):E613-E24.
3. Itz CJ, Geurts JW, van Kleef M, et al. Clinical course of non-specific low back pain: a systematic review of prospective cohort studies set in primary care. European journal of pain (London, England) 2013;**17**(1):5-15.
4. Qaseem A, Wilt TJ, McLean RM, et al. Noninvasive treatments for acute, subacute, and chronic low back pain: A clinical practice guideline from the american college of physicians. Annals of internal medicine 2017;**166**(7):514-30.
5. van Tulder M, Becker A, Bekkering T, et al. Chapter 3. European guidelines for the management of acute nonspecific low back pain in primary care. European spine journal : official publication of the European Spine Society, the European Spinal Deformity Society, and the European Section of the Cervical Spine Research Society 2006;**15 Suppl 2**:S169-91.
6. Chou R, Deyo R, Friedly J, et al. Systemic Pharmacologic Therapies for Low Back Pain: A Systematic Review for an American College of Physicians Clinical Practice Guideline. Annals of internal medicine 2017;**166**(7):480-92.
7. Chaparro LE, Furlan AD, Deshpande A, et al. Opioids compared with placebo or other treatments for chronic low back pain: an update of the Cochrane Review. Spine 2014;**39**(7):556-63.
8. Hoffman EM, Watson JC, St Sauver J, et al. Association of Long-term Opioid Therapy With Functional Status, Adverse Outcomes, and Mortality Among Patients With Polyneuropathy. JAMA Neurol 2017;**74**(7):773-79.
9. Wong JJ, Cote P, Sutton DA, et al. Clinical practice guidelines for the noninvasive management of low back pain: A systematic review by the Ontario Protocol for Traffic Injury Management (OPTIMa) Collaboration. European journal of pain (London, England) 2017;**21**(2):201-16.
10. Konijnenbelt-Peters J, van der Heijden C, Ekhart C, et al. Metamizole (Dipyrone) as an Alternative Agent in Postoperative Analgesia in Patients with Contraindications for Nonsteroidal Anti-Inflammatory Drugs. Pain practice : the official journal of World Institute of Pain 2017;**17**(3):402-08.
11. Andersohn F, Konzen C, Garbe E. Systematic review: agranulocytosis induced by nonchemotherapy drugs. Annals of internal medicine 2007;**146**(9):657-65.
12. Stammschulte T, Ludwig WD, Muhlbauer B, et al. Metamizole (dipyrone)-associated agranulocytosis. An analysis of German spontaneous reports 1990-2012. European journal of clinical pharmacology 2015;**71**(9):1129-38.
13. Hoffmann F, Meinecke P, Freitag MH, et al. Who gets dipyrone (metamizole) in Germany? Prescribing by age, sex and region. Journal of clinical pharmacy and therapeutics 2015;**40**(3):285-8.
14. Wertli MM, Reich O, Signorell A, et al. Changes over time in prescription practices of pain medications in Switzerland between 2006 and 2013: an analysis of insurance claims. BMC health services research 2017;**17**(1):167.
15. Hoffmann F, Bantel C, Jobski K. Agranulocytosis attributed to metamizole: An analysis of spontaneous reports in EudraVigilance 1985-2017. Basic & Clinical Pharmacology & Toxicology 2020;**126**(2):116-25.

For peer review only - http://bmjopen.bmj.com/site/about/guidelines.xhtml

16. Hoffmann F, Schmiemann G. Pain medication in German nursing homes: a whole lot of metamizole. Pharmacoepidemiology and drug safety 2016;**25**(6):646-51.
17. Kramer M, Lane D, Hutchinson T. Risks of agranulocytosis and aplastic anemia. A first report of their relation to drug use with special reference to analgesics. The International Agranulocytosis and Aplastic Anemia Study. Jama 1986;**256**(13):1749-57.
18. Huber M, Andersohn F, Sarganas G, et al. Metamizole-induced agranulocytosis revisited: results from the prospective Berlin Case-Control Surveillance Study. European journal of clinical pharmacology 2015;**71**(2):219-27.
19. Ibanez L, Vidal X, Ballarin E, et al. Population-based drug-induced agranulocytosis. Archives of internal medicine 2005;**165**(8):869-74.
20. Basak GW, Drozd-Sokolowska J, Wiktor-Jedrzejczak W. Update on the incidence of metamizole sodium-induced blood dyscrasias in Poland. The Journal of international medical research 2010;**38**(4):1374-80.
21. Maj S, Centkowski P. A prospective study of the incidence of agranulocytosis and aplastic anemia associated with the oral use of metamizole sodium in Poland. Medical science monitor : international medical journal of experimental and clinical research 2004;**10**(9):Pi93-5.
22. Ibanez L, Vidal X, Ballarin E, et al. Agranulocytosis associated with dipyrone (metamizol). European journal of clinical pharmacology 2005;**60**(11):821-9.
23. Shapiro S, Issaragrisil S, Kaufman DW, et al. Agranulocytosis in Bangkok, Thailand: a predominantly drug-induced disease with an unusually low incidence. Aplastic Anemia Study Group. The American journal of tropical medicine and hygiene 1999;**60**(4):573-7.
24. van der Klauw MM, Goudsmit R, Halie MR, et al. A population-based case-cohort study of drug-associated agranulocytosis. Archives of internal medicine 1999;**159**(4):369-74.
25. Hamerschlak N, Maluf E, Biasi Cavalcanti A, et al. Incidence and risk factors for agranulocytosis in Latin American countries--the Latin Study: a multicenter study. European journal of clinical pharmacology 2008;**64**(9):921-9.
26. Hamerschlak N, Cavalcanti AB. Neutropenia, agranulocytosis and dipyrone. Sao Paulo medical journal = Revista paulista de medicina 2005;**123**(5):247-9.
27. Maj S, Lis Y. The incidence of metamizole sodium-induced agranulocytosis in Poland. The Journal of international medical research 2002;**30**(5):488-95.
28. Varonos DD, Santamouris S, Karambali S. The incidence of dipyrone-induced agranulocytosis in Greece during 1975. The Journal of international medical research 1979;**7**(6):564-8.
29. Blaser LS, Tramonti A, Egger P, et al. Hematological safety of metamizole: retrospective analysis of WHO and Swiss spontaneous safety reports. European journal of clinical pharmacology 2015;**71**(2):209-17.
30. Kötter T, da Costa BR, Fässler M, et al. Metamizole-Associated Adverse Events: A Systematic Review and Meta-Analysis. PloS one 2015;**10**(4):e0122918.
31. Rosser BA, McCracken LM, Velleman SC, et al. Concerns about medication and medication adherence in patients with chronic pain recruited from general practice. Pain 2011;**152**(5):1201-5.
32. Engers A, Jellema P, Wensing M, et al. Individual patient education for low back pain. The Cochrane database of systematic reviews 2008(1):Cd004057.
33. Chan AW, Tetzlaff JM, Gøtzsche PC, et al. SPIRIT 2013 explanation and elaboration: guidance for protocols of clinical trials. BMJ (Clinical research ed) 2013;**346**:e7586.
34. Chow SC, Shao J, Wang H. *Sample Size Calculations in Clinical Research.* 2nd ed. New York: Chapman & Hall/CRC, 2008.

For peer review only - http://bmjopen.bmj.com/site/about/guidelines.xhtml

35. Farrar JT, Young JP, Jr., LaMoreaux L, et al. Clinical importance of changes in chronic pain intensity measured on an 11-point numerical pain rating scale. Pain 2001;**94**(2):149-58.
36. Ostelo RW, Deyo RA, Stratford P, et al. Interpreting change scores for pain and functional status in low back pain: towards international consensus regarding minimal important change. Spine 2008;**33**(1):90-4.
37. Mannion AF, Porchet F, Kleinstuck FS, et al. The quality of spine surgery from the patient's perspective: part 2. Minimal clinically important difference for improvement and deterioration as measured with the Core Outcome Measures Index. European spine journal : official publication of the European Spine Society, the European Spinal Deformity Society, and the European Section of the Cervical Spine Research Society 2009;**18 Suppl 3**:374-9.
38. Williams CM, Maher CG, Latimer J, et al. Efficacy of paracetamol for acute low-back pain: a double-blind, randomised controlled trial. Lancet (London, England) 2014;**384**(9954):1586-96.
39. Mannion AF, Elfering A, Staerkle R, et al. Outcome assessment in low back pain: how low can you go? European spine journal : official publication of the European Spine Society, the European Spinal Deformity Society, and the European Section of the Cervical Spine Research Society 2005;**14**(10):1014-26.
40. Pochon L, Kleinstuck FS, Porchet F, et al. Influence of gender on patient-oriented outcomes in spine surgery. European spine journal : official publication of the European Spine Society, the European Spinal Deformity Society, and the European Section of the Cervical Spine Research Society 2016;**25**(1):235-46.
41. Deyo RA, Dworkin SF, Amtmann D, et al. Report of the NIH Task Force on research standards for chronic low back pain. Pain medicine (Malden, Mass) 2014;**15**(8):1249-67.
42. Pincus T, Santos R, Breen A, et al. A review and proposal for a core set of factors for prospective cohorts in low back pain: a consensus statement. Arthritis Rheum 2008;**59**(1):14-24.
43. Karstens S, Krug K, Hill JC, et al. Validation of the German version of the STarT-Back Tool (STarT-G): a cohort study with patients from primary care practices. BMC musculoskeletal disorders 2015;**16**:346.
44. Weiser T, Schepers C, Mück T, et al. Pharmacokinetic Properties of Ibuprofen (IBU) From the Fixed-Dose Combination IBU/Caffeine (400/100 mg; FDC) in Comparison With 400 mg IBU as Acid or Lysinate Under Fasted and Fed Conditions—Data From 2 Single-Center, Single-Dose, Randomized Crossover Studies in Healthy Volunteers. Clinical Pharmacology in Drug Development 2019;**8**(6):742-53.
45. Pierre SC, Schmidt R, Brenneis C, et al. Inhibition of cyclooxygenases by dipyrone. British journal of pharmacology 2007;**151**(4):494-503.
46. García-Martín E, Martínez C, Tabarés B, et al. Interindividual variability in ibuprofen pharmacokinetics is related to interaction of cytochrome P450 2C8 and 2C9 amino acid polymorphisms. Clinical Pharmacology & Therapeutics 2004;**76**(2):119-27.
47. Kirchheiner J, Meineke I, Freytag G, et al. Enantiospecific effects of cytochrome P450 2C9 amino acid variants on ibuprofen pharmacokinetics and on the inhibition of cyclooxygenases 1 and 2. Clinical Pharmacology & Therapeutics 2002;**72**(1):62-75.
48. Waddell G, Newton M, Henderson I, et al. A Fear-Avoidance Beliefs Questionnaire (FABQ) and the role of fear-avoidance beliefs in chronic low back pain and disability. Pain 1993;**52**(2):157-68.

For peer review only - http://bmjopen.bmj.com/site/about/guidelines.xhtml

49. Mangels M, Schwarz S, Sohr G, et al. Der Fragebogen zur Erfassung der schmerzspezifischen Selbstwirksamkeit (FESS) D Miaganogsetlisc ea, 2009;**55**(2):84-93.
50. Mayring P. *Qualitative Inhaltsanalyse. Grundlagen und Techniken*. 7. ed. Weinheim: Deutscher Studien Verlag, 2000.
51. Klueglich M, Ring A, Scheuerer S, et al. Ibuprofen extrudate, a novel, rapidly dissolving ibuprofen formulation: relative bioavailability compared to ibuprofen lysinate and regular ibuprofen, and food effect on all formulations. Journal of clinical pharmacology 2005;**45**(9):1055-61.
52. Kyselovič J, Koscova E, Lampert A, et al. A Randomized, Double-Blind, Placebo-Controlled Trial of Ibuprofen Lysinate in Comparison to Ibuprofen Acid for Acute Postoperative Dental Pain. Pain and Therapy 2020;**9**(1):249-59.
53. Burton AK, Waddell G, Tillotson KM, et al. Information and advice to patients with back pain can have a positive effect. A randomized controlled trial of a novel educational booklet in primary care. Spine 1999;**24**(23):2484-91.
54. U.S. Department of health and human services. Common Terminology Criteria for Adverse Events (CTCAE). In: U.S.Department of health and human services NIoH, national Cancer Institute, ed. June 14, 2010 ed: U.S.Department of health and human services,, 2009.
55. Friedman BW, Dym AA, Davitt M, et al. Naproxen With Cyclobenzaprine, Oxycodone/Acetaminophen, or Placebo for Treating Acute Low Back Pain: A Randomized Clinical Trial. Jama 2015;**314**(15):1572-80.
56. Hagen KB, Hilde G, Jamtvedt G, et al. Bed rest for acute low-back pain and sciatica. The Cochrane database of systematic reviews 2004(4):Cd001254.
57. Gerfin A. Rückenreport Schweiz 2011: Die Erhebung zur Rückengesundheit von Herrn und Frau Schweizer. 2011 August 30, 2011. https://www.rheumaliga.ch/assets/doc/CH_Dokumente/medienmitteilungen/facts-and-figures/Rueckenreport-Schweiz-2011_Rheumaliga-Schweiz.pdf.

For peer review only - http://bmjopen.bmj.com/site/about/guidelines.xhtml

Figure Title and Legend

Figure 1. Study flow chart

For peer review only

For peer review only - http://bmjopen.bmj.com/site/about/guidelines.xhtml

Tables

Table 1: Summary of study procedures based on Standard Protocol Items: Recommendations for Interventional Trials (SPIRIT) schedule

	Enrollment	Allocation	Short intervention	Phone call	Clinical visit	Phone call	Clinical visit or phone call
**Time point (days)**	-5 to 0	0	2	7	14	28	42
Clinical visit	1	1			2		**3 or ✆**[4]
Phone call			✆[2]	✆[3]		✆[3]	
Patient information & informed consent	x						
In-/Exclusion	x						
Demographics	x						
Medical History	x						
Physical examination	x						
Vital signs	x				x		(x)
Blood analysis	x				x		(x)
Pregnancy test	x						
Randomization		x					
Hand out study medication		x					
Daily intake of study medication		x					
Group 1 + 3: study medication only		x		x	x	x	x
Group 2 + 4: study medication + short intervention		x	x	x	x	x	x
Pain diary daily including:<br>- Pain<br>- additional treatments<br>- AE<br>- Daily physical activity		x	x	x	x	x	x
Adherence reminder[3]				x		x	

For peer review only

For peer review only - http://bmjopen.bmj.com/site/about/guidelines.xhtml

Screening for: [9]<br>- AE and SAE<br>- interrupting or discontinuing treatment				x	x	x	x
Collection of pill bottles for pill-count					x		x
Self-reported measures collected during visits							
Pain questionnaire (NRS)		x			x		x
COMI		x			x		x
STarT Back tool		x			x		x
Quality of life (EQ5-D-5L)		x			x		x
Fear avoidance (FABQ)		x					
Self-efficacy (FESS)		x					
Blinding control							x
Voluntary in-person interview on patient perspective							x

Legend: AE, adverse event; SAE, serious adverse event; NRS, numeric rating scale; COMI, Core Outcome Measures Index; EQ5-D-5L, European Quality of Life-5 Dimensions questionnaire; FABQ, Fear Avoidance Questionnaire, FESS, self-efficacy (Schmerzspezifische Selbstwirksamkeit)

For peer review only

Table 2: Oral doses for study medication – fixed dose and as-needed regimen

Group	Study medication	Fixed dose regimen (days 0 - 3)	As-needed regimen (after day 3): recommended reduction of the oral intake when LBP decreases
1	Ibuprofen only	500mg§ 2-2-2	2-2-2 (pain unchanged), 1-1-1 (decreased pain; NRS <4 points), 1-0-1, 1-0-0
2	Ibuprofen + short intervention	500mg§ 2-2-2	2-2-2 (pain unchanged), 1-1-1 (decreased pain; NRS <4 points), 1-0-1, 1-0-0
3	Metamizole only	500mg 2-2-2	2-2-2 (pain unchanged), 1-1-1 (decreased pain; NRS <4 points), 1-0-1, 1-0-0
4	Metamizole + short intervention	500mg 2-2-2	2-2-2 (pain unchanged), 1-1-1 (decreased pain; NRS <4 points), 1-0-1, 1-0-0

§500 mg ibuprofenum lysinum (lysine-2-[4-isobutylphenyl]-propionate) corresponds to 292.6 mg ibuprofen acid.

For peer review only - http://bmjopen.bmj.com/site/about/guidelines.xhtml

Abbreviations

AE	Adverse Event
CDMS	Clinical Data Management System
COMI	Core Outcome Measures Index
CRF	Case Report Form
EMISI	Efficacy of Metamizole versus Ibuprofen and a Short Educational Intervention versus Standard Care
GP	General Physician
ITT	Intention To Treat
LBP	Low Back Pain
NRS	Numeric Rating Scale
NSAIDs	Non-Steroidal Anti-Inflammatory Drugs

For peer review only

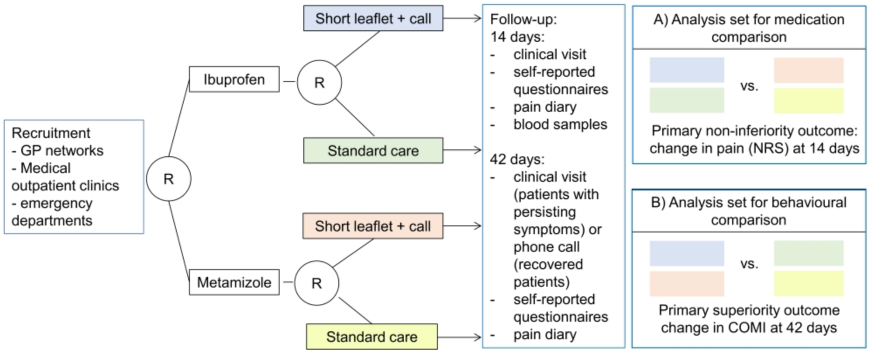

338x190mm (600 x 600 DPI)

Reporting checklist for protocol of a clinical trial.

Based on the SPIRIT guidelines.

Instructions to authors

Complete this checklist by entering the page numbers from your manuscript where readers will find each of the items listed below.

Your article may not currently address all the items on the checklist. Please modify your text to include the missing information. If you are certain that an item does not apply, please write "n/a" and provide a short explanation.

Upload your completed checklist as an extra file when you submit to a journal.

In your methods section, say that you used the SPIRITreporting guidelines, and cite them as:

Chan A-W, Tetzlaff JM, Gøtzsche PC, Altman DG, Mann H, Berlin J, Dickersin K, Hróbjartsson A, Schulz KF, Parulekar WR, Krleža-Jerić K, Laupacis A, Moher D. SPIRIT 2013 Explanation and Elaboration: Guidance for protocols of clinical trials. BMJ. 2013;346:e7586

		Reporting Item	Page Number
**Administrative information**			
Title	#1	Descriptive title identifying the study design, population, interventions, and, if applicable, trial acronym	1

For peer review only

Trial registration	#2a	Trial identifier and registry name. If not yet registered, name of intended registry	3
Trial registration: data set	#2b	All items from the World Health Organization Trial Registration Data Set	
Protocol version	#3	Date and version identifier	4
Funding	#4	Sources and types of financial, material, and other support	2, 24
Roles and responsibilities: contributorship	#5a	Names, affiliations, and roles of protocol contributors	23
Roles and responsibilities: sponsor contact information	#5b	Name and contact information for the trial sponsor	24
Roles and responsibilities: sponsor and funder	#5c	Role of study sponsor and funders, if any, in study design; collection, management, analysis, and interpretation of data; writing of the report; and the decision to submit the report for publication, including whether they will have ultimate authority over any of these activities	9
Roles and responsibilities: committees	#5d	Composition, roles, and responsibilities of the coordinating centre, steering committee, endpoint adjudication committee, data management team, and	9 and 17-19

For peer review only

		other individuals or groups overseeing the trial, if applicable (see Item 21a for data monitoring committee)	
**Introduction**			
Background and rationale	#6a	Description of research question and justification for undertaking the trial, including summary of relevant studies (published and unpublished) examining benefits and harms for each intervention	5-6
Background and rationale: choice of comparators	#6b	Explanation for choice of comparators	15-16
Objectives	#7	Specific objectives or hypotheses	6
Trial design	#8	Description of trial design including type of trial (eg, parallel group, crossover, factorial, single group), allocation ratio, and framework (eg, superiority, equivalence, non-inferiority, exploratory)	6-7
**Methods: Participants, interventions, and outcomes**			
Study setting	#9	Description of study settings (eg, community clinic, academic hospital) and list of countries where data will be collected. Reference to where list of study sites can be obtained	9

For peer review only

Eligibility criteria	#10	Inclusion and exclusion criteria for participants. If applicable, eligibility criteria for study centres and individuals who will perform the interventions (eg, surgeons, psychotherapists)	7-8
Interventions: description	#11a	Interventions for each group with sufficient detail to allow replication, including how and when they will be administered	15-17
Interventions: modifications	#11b	Criteria for discontinuing or modifying allocated interventions for a given trial participant (eg, drug dose change in response to harms, participant request, or improving / worsening disease)	14, 21-22
Interventions: adherance	#11c	Strategies to improve adherence to intervention protocols, and any procedures for monitoring adherence (eg, drug tablet return; laboratory tests)	14-17
Interventions: concomitant care	#11d	Relevant concomitant care and interventions that are permitted or prohibited during the trial	15-17
Outcomes	#12	Primary, secondary, and other outcomes, including the specific measurement variable (eg, systolic blood pressure), analysis metric (eg, change from baseline, final value, time to event), method of aggregation (eg, median, proportion), and time point for each outcome. Explanation of the clinical relevance of chosen efficacy and harm outcomes is strongly recommended	10-13

For peer review only

Participant timeline	#13	Time schedule of enrolment, interventions (including any run-ins and washouts), assessments, and visits for participants. A schematic diagram is highly recommended (see Figure)	Table 1, Figure 1
Sample size	#14	Estimated number of participants needed to achieve study objectives and how it was determined, including clinical and statistical assumptions supporting any sample size calculations	9-10
Recruitment	#15	Strategies for achieving adequate participant enrolment to reach target sample size	9, 13-15
**Methods: Assignment of interventions (for controlled trials)**			
Allocation: sequence generation	#16a	Method of generating the allocation sequence (eg, computer-generated random numbers), and list of any factors for stratification. To reduce predictability of a random sequence, details of any planned restriction (eg, blocking) should be provided in a separate document that is unavailable to those who enrol participants or assign interventions	17
Allocation concealment mechanism	#16b	Mechanism of implementing the allocation sequence (eg, central telephone; sequentially numbered, opaque,	17, 18

For peer review only

		sealed envelopes), describing any steps to conceal the sequence until interventions are assigned	
Allocation: implementation	#16c	Who will generate the allocation sequence, who will enrol participants, and who will assign participants to interventions	17
Blinding (masking)	#17a	Who will be blinded after assignment to interventions (eg, trial participants, care providers, outcome assessors, data analysts), and how	7, 16-18
Blinding (masking): emergency unblinding	#17b	If blinded, circumstances under which unblinding is permissible, and procedure for revealing a participant's allocated intervention during the trial	22
**Methods: Data collection, management, and analysis**			
Data collection plan	#18a	Plans for assessment and collection of outcome, baseline, and other trial data, including any related processes to promote data quality (eg, duplicate measurements, training of assessors) and a description of study instruments (eg, questionnaires, laboratory tests) along with their reliability and validity, if known. Reference to where data collection forms can be found, if not in the protocol	10-15, 17

Data collection plan: retention	#18b	Plans to promote participant retention and complete follow-up, including list of any outcome data to be collected for participants who discontinue or deviate from intervention protocols	9, 13-15
Data management	#19	Plans for data entry, coding, security, and storage, including any related processes to promote data quality (eg, double data entry; range checks for data values). Reference to where details of data management procedures can be found, if not in the protocol	19
Statistics: outcomes	#20a	Statistical methods for analysing primary and secondary outcomes. Reference to where other details of the statistical analysis plan can be found, if not in the protocol	19-21
Statistics: additional analyses	#20b	Methods for any additional analyses (eg, subgroup and adjusted analyses)	19-21
Statistics: analysis population and missing data	#20c	Definition of analysis population relating to protocol non-adherence (eg, as randomised analysis), and any statistical methods to handle missing data (eg, multiple imputation)	19-21

Methods: Monitoring

Data monitoring: formal committee	#21a	Composition of data monitoring committee (DMC); summary of its role and reporting structure; statement of whether it is independent from the sponsor and	24

For peer review only

		competing interests; and reference to where further details about its charter can be found, if not in the protocol. Alternatively, an explanation of why a DMC is not needed	
Data monitoring: interim analysis	#21b	Description of any interim analyses and stopping guidelines, including who will have access to these interim results and make the final decision to terminate the trial	24
Harms	#22	Plans for collecting, assessing, reporting, and managing solicited and spontaneously reported adverse events and other unintended effects of trial interventions or trial conduct	12, 14-15, 21-22
Auditing	#23	Frequency and procedures for auditing trial conduct, if any, and whether the process will be independent from investigators and the sponsor	24
**Ethics and dissemination**			
Research ethics approval	#24	Plans for seeking research ethics committee / institutional review board (REC / IRB) approval	23
Protocol amendments	#25	Plans for communicating important protocol modifications (eg, changes to eligibility criteria, outcomes, analyses) to relevant parties (eg, investigators, REC / IRBs, trial participants, trial registries, journals, regulators)	23

For peer review only

Consent or assent	#26a	Who will obtain informed consent or assent from potential trial participants or authorised surrogates, and how (see Item 32)	14
Consent or assent: ancillary studies	#26b	Additional consent provisions for collection and use of participant data and biological specimens in ancillary studies, if applicable	14
Confidentiality	#27	How personal information about potential and enrolled participants will be collected, shared, and maintained in order to protect confidentiality before, during, and after the trial	23
Declaration of interests	#28	Financial and other competing interests for principal investigators for the overall trial and each study site	24
Data access	#29	Statement of who will have access to the final trial dataset, and disclosure of contractual agreements that limit such access for investigators	23
Ancillary and post trial care	#30	Provisions, if any, for ancillary and post-trial care, and for compensation to those who suffer harm from trial participation	24
Dissemination policy: trial results	#31a	Plans for investigators and sponsor to communicate trial results to participants, healthcare professionals, the public, and other relevant groups (eg, via publication, reporting in results databases, or other data sharing arrangements), including any publication restrictions	24

For peer review only

Dissemination policy: authorship	#31b	Authorship eligibility guidelines and any intended use of professional writers	24
Dissemination policy: reproducible research	#31c	Plans, if any, for granting public access to the full protocol, participant-level dataset, and statistical code	24
**Appendices**			
Informed consent materials	#32	Model consent form and other related documentation given to participants and authorised surrogates	-
Biological specimens	#33	Plans for collection, laboratory evaluation, and storage of biological specimens for genetic or molecular analysis in the current trial and for future use in ancillary studies, if applicable	12-13

None The SPIRIT checklist is distributed under the terms of the Creative Commons Attribution License CC-BY-ND 3.0. This checklist can be completed online using https://www.goodreports.org/, a tool made by the EQUATOR Network in collaboration with Penelope.ai

For peer review only
